# Development of highly efficient Friedel–Crafts alkylations with alcohols using heterogeneous catalysts under continuous-flow conditions†

**DOI:** 10.1039/d1ra04005g

**Published:** 2021-07-13

**Authors:** Koichiro Masuda, Yukiko Okamoto, Shun-ya Onozawa, Nagatoshi Koumura, Shū Kobayashi

**Affiliations:** Interdisciplinary Research Center for Catalytic Chemistry, National Institute of Advanced Industrial Science and Technology (AIST) 1-1-1 Higashi Tsukuba Ibaraki 305-8565 Japan koichiro-masuda@aist.go.jp shu_kobayashi@chem.s.u-tokyo.ac.jp; Department of Chemistry, School of Science, The University of Tokyo 7-3-1 Hongo Bunkyo-ku Tokyo 113-0033 Japan

## Abstract

The development of Friedel–Crafts alkylations with alcohols under continuous-flow conditions using heterogeneous catalysts is reported. The reactivities and durabilities of the examined catalysts were systematically investigated, which showed that montmorillonite clay is the best catalyst for these reactions. A high turnover frequency of 9.0 × 10^2^ h^−1^ was recorded under continuous-flow conditions, and the continuous operation was successfully maintained over one week.

## Abstract 2

Efficient and highly durable catalysts were developed for Friedel–Crafts alkylation using alcohols under continuous flow conditions.

## Graphical abstract

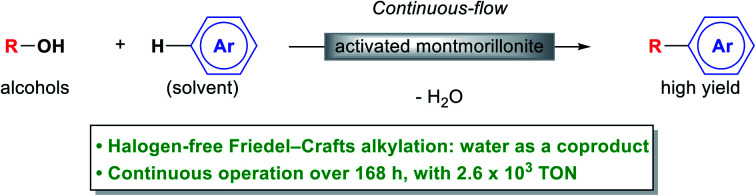

Efficient and high-throughput synthetic methods are in high demand for the ideal continuous production of fine chemicals. Continuous-flow syntheses, especially using packed bed reactors, offer green and sustainable methods that yield desired materials as sole products,^1^ and the development of highly active and durable heterogeneous catalysts is an important task in this research area.

Aromatic alkylations, in particular, the Friedel–Crafts alkylation, are among the most employed organic reactions,^2^ with classical processes using alkyl halides and aromatic compounds in the presence of acids as promoters. Alkyl halides are to be avoided from the viewpoint of green and sustainable chemistry, as these substrates yield hydrogen halides as coproducts, which are not only corrosive but also autocatalytic, boosting reactivity and often resulting in uncontrollable reactions.

Alcohols are environmentally benign alternatives.^2*b*^ While the methylation of toluene with methanol is a well-known industrial reaction, within the process window of fine chemicals, Friedel–Crafts alkylations with alcohols suffer from low reactivities and catalyst deactivation. Alcoholic hydroxyl groups and water, the coproduct of the reaction, can destroy catalytic activity and even hydrolyse catalysts to inert structures. Recent water-tolerant Lewis acid^3^ and heterogeneous acid catalyst developments have enabled the catalytic conversions of activated alcohols (and aldehydes) with solvent amounts of arenes.^4^ Supported iron oxide,^5^ a fluorinated polymer-supported Brønsted acid,^6^ and hydroxyl-functionalised sulfonic acid silica^7^ were used as fixed bed catalysts to achieve continuous-flow reactions with considerably high turnover frequencies (TOFs).

In this study, we systematically evaluated a series of heterogeneous acid catalysts for Friedel–Crafts alkylations with alcohols and the excellent catalytic activities of layered aluminosilicates.

The reaction of benzyl alcohol (1a) with *p*-xylene was chosen as the model system (Scheme 1). Under acidic conditions, 1a provided Friedel–Crafts product 2a and dimeric ether 3a; further reaction of 3a furnished 2a. Self-oligomerisation of 1a, initiated by overalkylation of any aromatic ring, but mainly those in 2a, is a possible side reaction of this system,^8^ as confirmed by the strong fluorescence of polybenzyl structures.^9^ Therefore, catalysts were evaluated and discussed using the conversion of 1a in relation to selectivity for 2a and 3a. The catalysts and their conditions are summarised in Table 1. Column size, catalyst loading, and flow rate were varied to achieve optimal conditions. It is necessary that the whole catalyst in the column is effective for easier comparison of the catalyst performance.^10^ The results in reactors of varying size under various conditions are summarised by weight hourly space velocity (WHSV) and substrate/catalyst weight ratio (S/C). The progress of each flow reaction, initially obtained as conversion-time data, is plotted against S/C ratio for normalisation, as shown in Fig. 1.

**Scheme 1:**
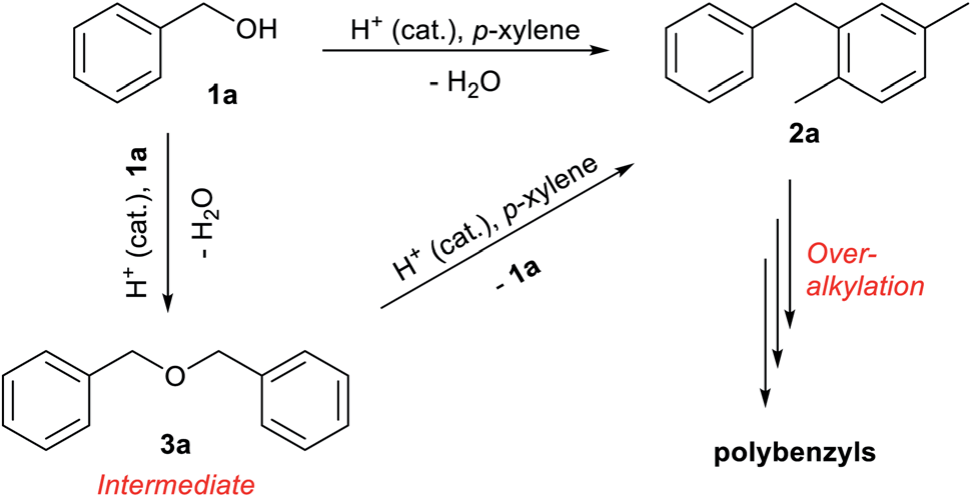
Acid-catalysed reactions of benzyl alcohol (1a) in *p*-xylene.

**Table 1:** Evaluation of heterogeneous acid catalysts^a^

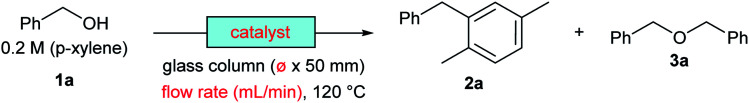						
Entry	Catalyst	Column (mm)	Flow rate (mL min^−1^)	Reaction scale	WHSV^b^ (h^−1^)	Average selectivity^c^
1	H-Y zeolite (501.5 mg)	ø5 × L50	0.05	10 mmol (50 mL)	0.13	83
2	H-beta zeolite (537.7 mg)	ø5 × L50	0.05	10 mmol (50 mL)	0.12	82
3	Nafion/silica (338.9 mg)	ø5 × L50	0.05	10 mmol (50 mL)	0.19	90
4	Sulf. ZrO_2_ (1349 mg)	ø5 × L50	0.25	20 mmol (100 mL)	0.24	85
5	Mont. K10^d^ (47.5 mg)	ø5 × L50	0.10	20 mmol (100 mL)	2.7	85
6	Bentonite^d^ (18.7 mg)	ø3 × L50	1.00	20 mmol (100 mL)	69	77
7	H-mont.^d^ (16.6 mg)	ø3 × L50	1.00	20 mmol (100 mL)	78	76

a Procedure: each catalyst was packed into a glass column for an EYELA MCR-1000 column-type flow reactor and pre-treated with *p*-xylene at the designed flow rate until all inside air has been ejected. A solution of 1a in *p*-xylene (0.2 M, 10 or 20 mmol scale with 0.04 M pentadecane as the internal standard) was pumped into the column heated at 120 °C, and the obtained mixture was fractionated into test tubes for analysis.

b Average selectivity over whole fraction collected toward 2a and 3a, calculated as (2a + 2 × 3a)/(conversion of 1a).

c The catalyst was diluted with celite (1/9).

d WHSV describes the relative substrate mass feed speed to the catalyst, and defined as follows: WHSV = [flow rate (mL min^−1^)] × [Conc. (g L^−1^)] × 60 (min h^−1^)/*W*_catalyst_.

**Fig. 1:**
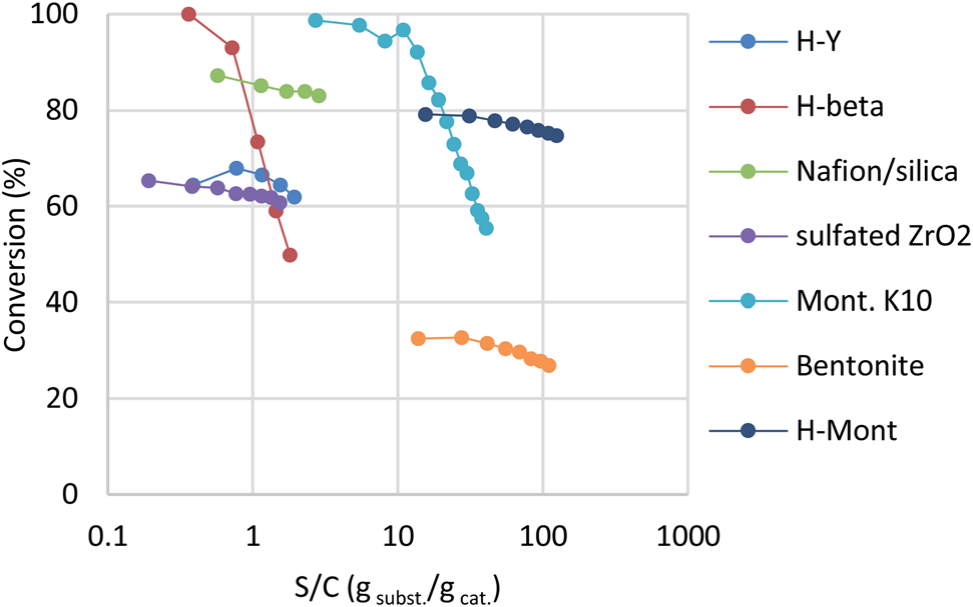
Evaluation of heterogeneous acid catalysts. The results are given as conversion *vs.* S/C plots; semi-log forms were employed due to wide variations on the S/C axis. S/C is given as the integration of WHSV over time, with the value calculated as follows, assuming constant flow rate: S/C = WHSV × *t*.

Zeolites have been reportedly used in Friedel–Crafts alkylation chemistry with benzyl alcohols under batch conditions.^4*b*,11^ Among various structures, H-Y and H-beta zeolites were chosen as typical acid catalysts in this study. While H-Y showed moderate reactivity at a flow rate of 0.05 mL min^−1^, H-beta recorded full conversion at the beginning (Table 1, entries 1 and 2; blue and red in Fig. 1). Interestingly, the activity of H-beta decreased more rapidly than that of H-Y, showing good linearity in the semi-log plot.

Nafion-H has been reported to be a good catalyst for this reaction under batch conditions.^12^ The low reactivity reported under flow conditions^6^ was assumed to be due to the small surface area of the commercial Nafion NR50 solid; therefore, we immobilised Nafion DE521 dispersion on silica gel^13^ and then applied it to the reaction, which led to high selectivity and more-stable catalytic activity than those observed for the zeolites (entry 3; right green). Sulfated zirconia is also known to be a strong heterogeneous Brønsted acid;^14^ this material was found to be a good catalyst for this reaction, with stable reactivity and selectivity observed (entry 4, purple). Metal-exchanged clays^15^ or acidified ones^16^ have been reported to be good catalysts for the activation of benzyl alcohols under batch conditions; we found that commercial montmorillonite K10 was highly efficient for this reaction (entry 5, turquoise). This clay catalyst required dilution with celite to avoid column stacking, despite its considerably high reactivity. Moreover, bentonite freshly activated with 1 M sulfuric acid at room temperature (a raw clay mineral containing montmorillonite as the main component provided by Fujifilm Wako Chemicals) showed excellent activity (entry 6, orange). It should be noted that an experiment was conducted with a more than 25-times larger WHSV, a reduced column volume of 36%, and a 10-times faster flow rate owing to the extremely high catalytic activity of this clay. Interestingly, while a significant drop in activity was observed using commercial montmorillonite K10, house-acidified natural clay maintained its catalytic efficiency even after processing large amounts of substrate. The differences between the two clays prompted us to conduct further investigations. Purified montmorillonite (Kunipia F, provided by Kunimine Industry), acidified under the same conditions as the bentonite catalyst, exhibited excellent productivity and durability (entry 7, deep blue).

In a previous report,^16^ montmorillonite was activated with dilute HCl, whereas commercial K10 used sulfuric acid as the activating reagent. However, our trials showed that aqueous HCl treatment resulted in slightly lower catalytic activity than sulfuric acid (Table 2, entry 1; purple plot in Fig. 2). Interestingly, concentrated HCl (35%) provided a higher conversion and better catalyst stability (entry 2, turquoise). In order to observe clear catalyst differences, montmorillonites treated with concentrated HCl and 1 M HCl were re-examined under further accelerated conditions (entries 3 and 4, orange and deep blue). A ten-fold-diluted catalyst column was employed at a slower flow rate to ensure that the experiment was conducted in the higher turnover region without reaction scale-up. The concentrated-HCl-treated catalyst showed a slower drop in catalyst activity, which indicates that conc. HCl treatment is optimal for catalyst preparation (entry 4). Under these conditions, the reaction provided a turnover number (TON) of 1.09 × 10^4^ for a 20 mmol-scale experiment, with a turnover frequency (TOF) of 9.0 × 10^2^ h^−1^ at the initial stage of the flow reaction. With this catalyst in hand, standard synthetic conditions were established by reducing the WHSV to 6.8 (ø5 × 50 mm column with 1/9 catalyst dilution, 0.25 mL min^−1^), which achieved full conversion with minimal amounts of intermediate 3a detected (see ESI† for details). These synthetic conditions delivered a TON of 491 and a TOF of 76.7 h^−1^ based on conversion, with 2.28 mmol h^−1^ productivity of the desired product and a 456 g h^−1^ dm^−3^ space time yield (STY). All fractions were collected, with the desired product isolated in 63% yield.

**Table 2:** Fine-tuning montmorillonite under accelerated conditions^a^

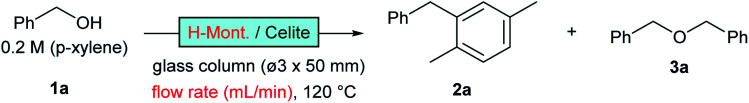						
Entry	Acid treatment	Cat/celite	Catalyst loading (mg)	Flow rate (mL min^−1^)	WHSV (h^−1^)	Average selectivity^b^
(Table 1, entry 7)	H_2_SO_4_ (1 M)	1/9	16.6	1.0	78	76%
1	HCl (1 M)	1/9	16.4	1.0	79	75%
2	HCl (conc.)	1/9	16.1	1.0	81	77%
3	HCl (1 M)	1/99	1.92	0.1	68	73%
4	HCl (conc.)	1/99	3.36	0.25	97	70%

a Procedure: the catalyst was mixed with Celite® and packed into a glass column for an EYELA MCR-1000 column-type flow reactor and pre-treated with *p*-xylene at the designed flow rate until all inside air had been ejected. A solution of 1a in *p*-xylene (0.2 M, 20 mmol scale with 0.04 M pentadecane as the internal standard) was pumped into a column heated at 120 °C, and the obtained mixture was fractionated into test tubes for analysis.

b Average selectivity toward 2a and 3a, calculated as (2a + 2 × 3a)/(conversion of 1a).

**Fig. 2:**
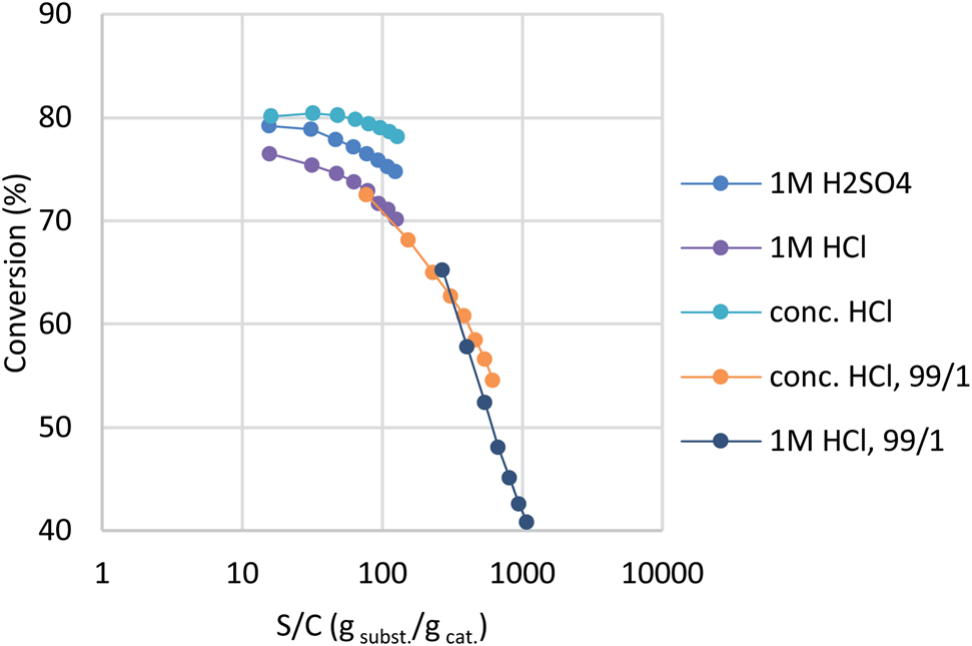
Fine-tuning montmorillonite under accelerated conditions.

The scope and limitations of the reaction were investigated under the established synthetic conditions (Scheme 2). The reaction was initially examined with benzyl alcohol as the substrate in benzene, toluene, and anisole as solvents. The desired products 2c and 2d were obtained successfully in toluene and anisole, while column clogging was observed within 1 h in benzene; the formation of polybenzyl seems to be a problem. We assume that the less reactive benzene is unable to compete with the overalkylation of any alkylated product, resulting in uncontrollable polymerisation that clogged the reactor. A similar tendency was observed for toluene; the desired product 2c was obtained, however, yield and selectivity were not comparable with those of *p*-xylene, and the column pressure gradually increased during the flow reaction. More nucleophilic anisole, as solvent, provided good reactivity, with the desired product 2d obtained in good yield without any clogging observed. These monosubstituted aromatics gave *para*- and *ortho*-substituted products, with slightly *p*-favoured tendencies, presumably due to steric hindrance.

**Scheme 2:**
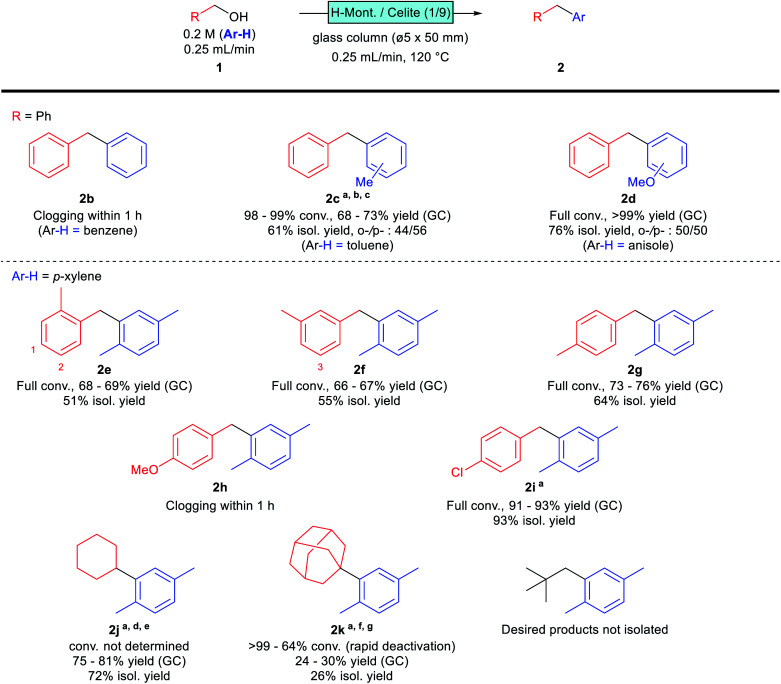
Substrate scope. Reactions were conducted according to the general procedure (see ESI†) unless otherwise noted. ^*a*^0.1 mL min^−1^. ^*b*^A ø5 × 100 (mm) column was used. ^*c*^Dodecane was used as the internal standard. ^*d*^A ø10 × 50 (mm) column was used. ^*e*^160 °C. ^*f*^0.1 M, 10 mmol scale. ^*g*^A ø10 × 100 (mm) column was used.

Monosubstituted benzyl alcohols were next investigated using *p*-xylene as the solvent. The position of the substituent affected the reactivity; *o*- and *m*-methylbenzyl alcohols gave the desired products 2e and 2f in moderate yields, while *p*-methylbenzyl alcohol afforded the desired 2g in a slightly higher yield. This difference is attributable to the steric hindrance of the product; 2e and 2f are less sterically hindered than 2g (Scheme 2, indicated as positions 1–3 in the structures). Therefore, 2e and 2f are more prone to overreacting with the substrate, which reduces their yields. Indeed, an increase in column pressure was observed at the end of the flow reaction involving *o*-methylbenzyl alcohol (1e), suggesting that this substrate has a higher tendency to polymerise. The reaction of the more electron-donating *p*-methoxybenzyl alcohol suffered from clogging, and the desired product 2h was not obtained. On the other hand, the electron-withdrawing *p*-chlorobenzyl alcohol reacted successfully, albeit with slightly lower reactivity, and required a smaller WHSV for reaction completion. The desired product 2i was obtained in high yield without any drop in reactivity or column clogging observed.

Several aliphatic alcohols were found to react under slightly modified conditions. Cyclohexanol provided the desired product 2j in good yield at higher temperatures and longer retention times, presumably *via* dehydrated cyclohexene as an intermediate. Conversion was not determined for this substrate, due to overlap with the solvent peak under standard GC conditions. 1-Adamantanol, reportedly a good aliphatic substrate,^7^ was found to be troublesome in our case, with rapid catalyst deactivation observed, especially at high temperatures; the desired 2k was isolated in only 26% yield after optimising the reaction conditions. Neopentyl alcohol gave no isolable amount of the desired product, which is presumably due to the instability of the primary carbocation intermediate.

To demonstrate the power of our continuous-flow reaction, *p*-chlorobenzyl alcohol was employed under long-term operation conditions (Fig. 3), which were slightly modified for stability. The desired product 2i was obtained in excellent yield without any loss of reactivity over one week (= 168 h), with an overall TON of 2.6 × 10^3^ recorded. All reaction fractions were combined and distilled to obtain pure 2i in 93% yield (91.2 g).

**Fig. 3:**
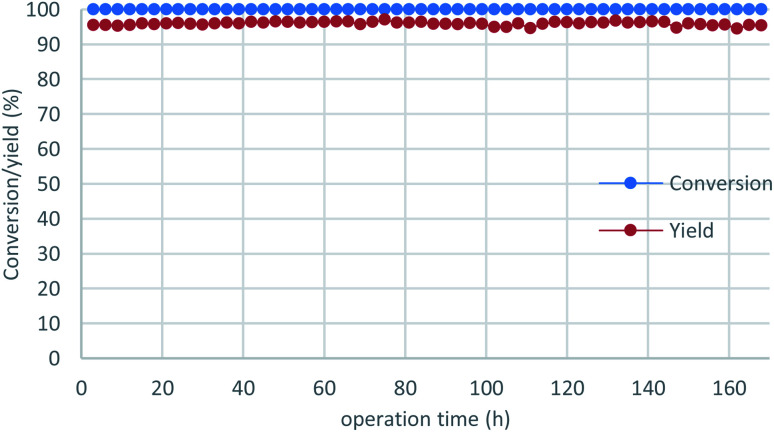
Long-term operation with *p*-chlorobenzyl alcohol (1i). The reaction was conducted with a ø10 × 100 mm column at a flow rate of 0.2 mL min^−1^. A total of 60.1 g of substrate was processed with this column at a catalyst loading of 187 mg.

In conclusion, a highly active and durable catalyst suitable for continuous flow synthesis was successfully developed for Friedel–Crafts alkylations using alcohols. Systematically investigating catalysts under continuous-flow conditions revealed clear differences in activity and durability, and identified activated montmorillonite clay as the best catalyst for these reactions. Alkylated products were obtained in moderate-to-high yields with some amounts of polymeric by-products, which could be controlled by adjusting the electronic properties of both the electrophile and nucleophile. *p*-Chlorobenzyl alcohol was employed in a long-term process, with the desired 2i produced almost quantitatively and isolated in 93% yield. Further studies into this continuous-flow chemistry are currently underway.

## Author contributions

KM contributed to conceptualising the project, evaluating the catalysts, and manuscript writing. YO investigated substrate scope and optimised the conditions. SO was involved in study conceptualisation. NK supervised the team and SK administered the entire project.

## Conflicts of interest

There are no conflicts to declare.

## Supplementary Material

RA-011-D1RA04005G-s001
