# Supplementary material for: Development of highly efficient Friedel–Crafts alkylations with alcohols using heterogeneous catalysts under continuous-flow conditions
Source: RSC Adv. 2021 Jul 13;11(39):24424–8. doi: 10.1039/d1ra04005g (PMC9036667; doi:10.1039/d1ra04005g)
Supplement: RA-011-D1RA04005G-s001 [file RA-011-D1RA04005G-s001.pdf]

Electronic Supplementary Material (ESI) for RSC Advances.
This journal is © The Royal Society of Chemistry 2021

Supplementally Information

Development of highly efficient heterogeneous catalysts for Friedel-Crafts alkylations with alcohols under continuous-flow conditions

Koichiro Masuda,*[a] Yukiko Okamoto,[a] Shun-ya Onozawa,[a] Nagatoshi Koumura[a] and Shū Kobayashi[a,b]

[a] Interdisciplinary Research Center for Catalytic Chemistry, National Institute of Advanced Industrial Science and Technology (AIST), 1-1-1 Higashi, Tsukuba, Ibaraki 305-8565, Japan

[b] Department of Chemistry, School of Science, The University of Tokyo, 7-3-1 Hongo, Bunkyo-ku, Tokyo 113-0033, Japan

Table of Contents

Experimental section 2
General 2
Representative procedures 2
Catalyst preparation 2
Continuous flow reaction 3
Compound characterisation 5
References 7
Reaction profiles 8
Table 1 8
Table 2 10
Substrate scope (scheme 2) 11
NMR spectra 15

Experimental section

General

^{1}H and ^{13}C NMR spectra were recorded on JEOL ECX400 spectrometer in $CDCl_3$ unless otherwise noted. Tetramethylsilane (TMS) served as an internal standard (δ = 0) for ^{1}H NMR, and $CDCl_3$ served as an internal standard (δ = 77.16) for ^{13}C NMR. IR analysis was conducted on JASCO FT/IR-6800 spectrometer equipped with ATR PRO550S-S diamond probe. High resolution mass spectra (HRMS) were obtained on a JEOL JMS-700. Gas chromatography was measured on a Shimadzu GC-2014 spectrometer with N_2 gas as a career, using Agilent DB-1 column (Length: 30 m, I.D.: 0.250 mm, Film: 0.25 µm). Energy dispersive X-ray fluorescence spectroscopy was measured on a Shimadzu EDX-8000 spectrometer. Powder XRD spectroscopy was measured on Rigaku MiniFlex 600. A dual plunger pump (UI-22 series) was purchased from FLOM, Inc., and flow reactor (MCR-1000) and fraction collector (DC-1000) were purchased from Tokyo Rikakikai Co., Ltd. H-Y and H-beta zeolites were obtained from Tosoh corp. Purified Montmorillonite (Kunipia F) was provided from Kunimine Industries Co., Ltd. Other chemicals and solvents were purchased from Tokyo Chemical Industry Co., Ltd, FUJIFILM Wako pure chemicals, Kishida chemical Co., Ltd., and Sigma-Aldrich.

Representative procedures

Catalyst preparation

Zeolites were used after calcination at 500 °C for 4 h under air. Sulfated zirconia (obtained from FUJIFILM Wako chemicals) and Montmorillonite K10 (obtained from Sigma-Aldrich) were used as received. Nafion/silica catalyst, Bentonite and H-Mont catalysts were prepared as follows.

- **Nafion/silica**

The title catalyst was prepared following reported method with slight modification.[1] To a dispersion of 3.00 g silica gel (CARiACT Q-15, 75 – 150 µm, Fuji Silysia chemical Ltd.) in 10 mL deionized water, 3.0 mL of 5wt% Naifon dispersion solution (DE521 CS type, FUJIFILM Wako pure chemicals) was added and the mixture was sonicated for 10 min. The mixture was evaporated under N2 flow at 60 °C until complete dryness. The material was then washed with 1N HCl and deionized water and dried again under vacuum at 100 °C to afford 3.10 g of desired catalyst. Catalyst loading was estimated to be 0.046 mmol/g from the amount of Nafion used and its reported acid capacity (>0.92 meq/g).

- **Acidification of Bentonite and purified montmorillonite**

2.16 g of purified Montmorillonite (Kunipia F) was dispersed in 200 mL of aqueous HCl (35wt%) and resulted mixture was kept still for 3 h. The mixture was poured into 2000 mL of MeOH and filtered. Extensive MeOH wash of filter cake and vacuum dryness afforded desired acidified Montmorillonite (H-Mont) in 1.94 g. Elemental analysis was conducted by EDX spectroscopy with fundamental parameter (FP) method; starting material (Na: 0.553%, Mg: 1.038%, Al: 8.849%, Si: 24.108%, Ca: 0.345%, Fe: 1.167%) and prepared catalyst (Na: ND, Mg: 1.494%, Al: 12.089%, Si: 34.04%, Ca: 0.006%,

Fe: 1.65%). Total acid site was determined to be 0.84 mmol/g by pyridine titration in hexane.
Powder XRD spectroscopy:

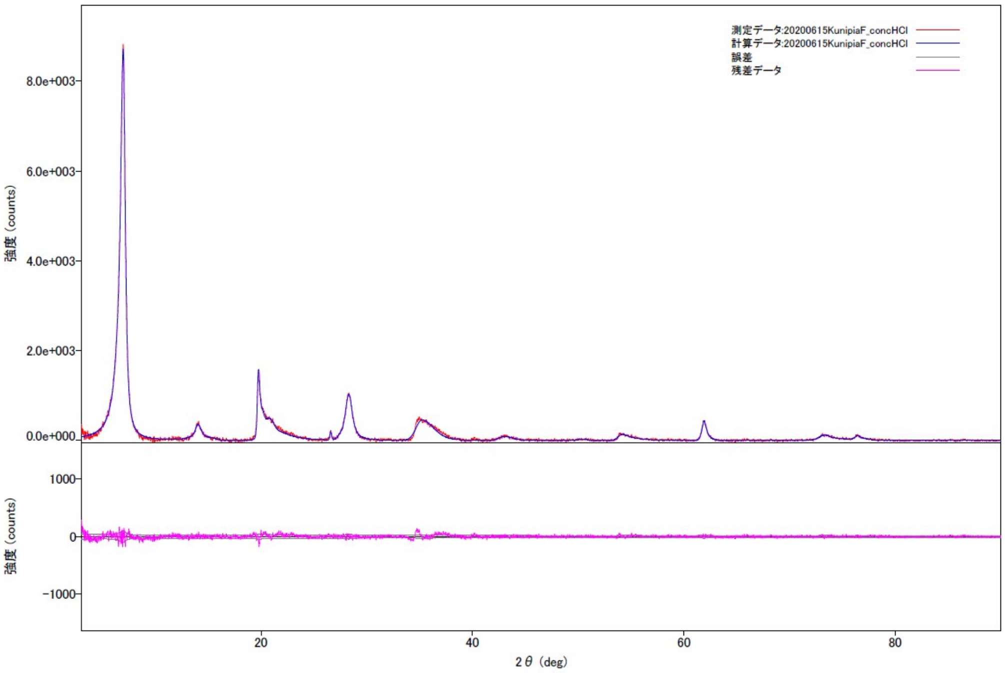

Continuous flow reaction

All the continuous flow experiment was conducted with MCR-1000 flow reactor with UI-22-110P dual plunger pump, equipped with fraction collector DC-1000 (See images below). **Standard synthetic conditions**: A H-Mont catalyst was pre-mixed with celite (catalyst/celite = 1/9) and then packed into a glass column (I.D. ø5 x L 50 mm, 477 mg; 47.7 mg for H-Mont) with PTFE filter. The catalyst cartridge was set onto the reactor, and *p*-xylene as a blank solvent was flowed at designated flow rate (0.25 mL/min) for 30 min to remove residual air inside. After wetting weight of cartridge was measured to confirm a volume of void space. The catalyst was heated at 120 °C with *p*-xylene flow, and after stabilization the reaction was started by changing into feed solution (100 mL, Benzyl alcohol **1a** (20 mmol, 0.2 M) with pentadecane (4 mmol, 0.04 M) as an internal standard) in *p*-xylene. The resulted solution was fractionated for every 24 min (6 mL), and selected fractions were analysed by GC-FID (N_2: 34.0 cm/s, column temperature: 50 °C for 2min, 20 °C/min heating then 250 °C for 10 min) to determine GC conversion and yield using pentadecane as an internal standard. After feed solution was finished blank solvent was flowed again, and all the product containing fractions were collected. After evaporation of solvent the product was isolated over column chromatography (hexane) to result desired product **2a** (2.48 g, 12.6 mmol, 63% yield).

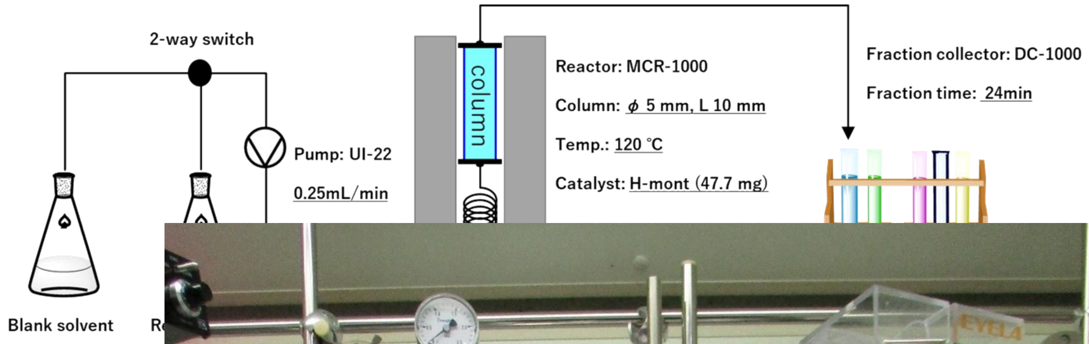

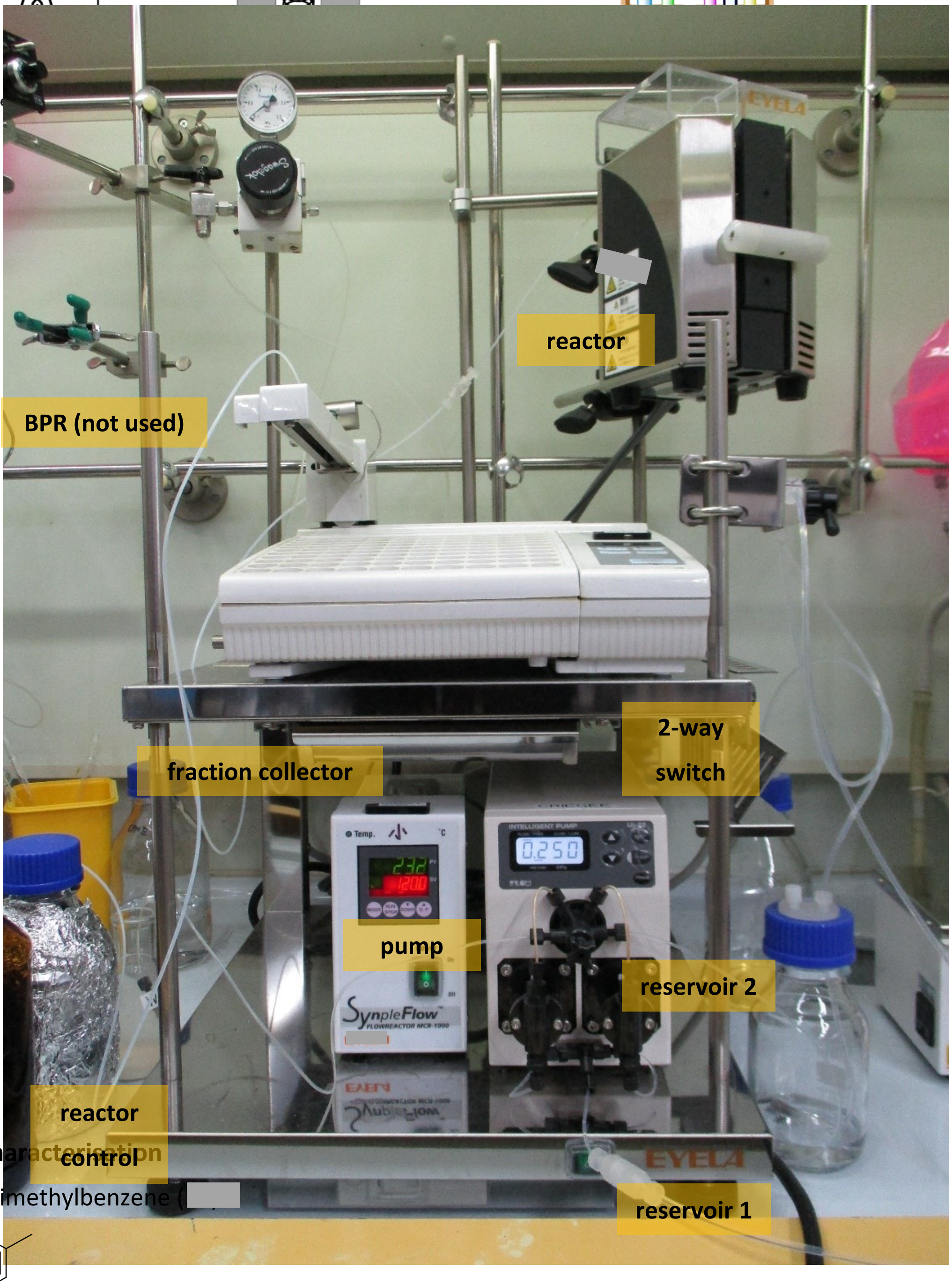

Compound characterization

1-benzyl-2,5-dimethylbenzene ([illegible])

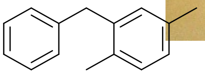

2.48 g (12.6 mmol, 63% yield) from 20 mmol scale experiment[illegible] δ_H (400 MHz, $CDCl_3$) 7.26 (2 H, t, *J* 7.4), 7.17 (1 H, t, *J* 7.3), 7.12 (2 H, d, *J* 7.0), 7.05 (1 H, d, *J* 7.7), 6.96 (1 H, d, *J* 7.7), 6.93 (1 H, s), 3.95

(2 H, s), 2.28 (3 H, s), 2.19 (3 H, s); δ_C (101 MHz, $CDCl_3$) 140.66, 138.77, 135.47, 133.54, 130.87, 130.28, 128.79, 128.44, 127.19, 125.94, 39.51, 21.07, 19.28.

o-benzyltoluene and *p*-benzyltoluene (**2c**), obtained as an inseparable mixture[2]

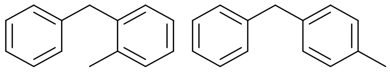

2.24 g (12.29 mmol, 61.4%, *o*- & *p*- mixture) from 20 mmol scale experiment. Dodecane was used as an internal standard instead of pentadecane due to its peak overlap. Regioisomer ratio was determined to be 44/56 (*o*-/*p*-) from crude NMR analysis. δ_H (400 MHz, $CDCl_3$) 7.32 – 7.21 (4.8 H, m, *o*- and *p*-), 7.21 – 7.04 (15.6 H, m, *o*- and *p*-), 3.98 (2 H (relative standard), s, *o*-), 3.94 (2.5 H, s, *p*-), 2.31 (3.7 H, s, *p*-), 2.24 (3 H, s, *o*-); δ_C (101 MHz, $CDCl_3$) 141.6 (*p*-), 140.5 (*o*-), 139.1 (*o*-), 138.2 (*p*-), 136.8 (*o*-), 135.7 (*p*-), 130.4 (*o*-), 130.1 (*o*-), 129.3 (*p*-), 129.0 (*p*-), 128.93 (*o*-), 128.86 (*p*-), 128.6 (*p*-), 128.5 (*o*-), 126.6 (*o*-), 126.1 (*o*-), 126.0 (*p*-), 41.6 (*p*-), 39.6 (*o*-), 21.1 (*p*-), 19.8 (*o*-).

o-benzylanisole and *p*-benzylanisole (**2d**)

1.51 g (*o*-) and 1.51 g (*p*-), totally 3.02 g (15.23 mmol, 76.2%) from 20 mmol scale experiment. Regioisomer ratio was determined to be 50/50 from crude NMR analysis. Isomers were successfully separated by column chromatography.

o-benzylanisole (**2d**)[3]

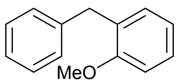

δ_H (400 MHz, $CDCl_3$) 7.30 – 7.12 (6 H, m), 7.06 (1 H, dd, *J* 7.8, 1.7), 6.91 – 6.81 (2 H, m), 3.97 (2 H, s), 3.81 (3 H, s); δ_C (101 MHz, $CDCl_3$) 157.4, 141.1, 130.3, 129.7, 129.0, 128.3, 127.4, 125.8, 120.5, 110.4, 55.4, 35.9.

p-benzylanisole (**2d**)[3]

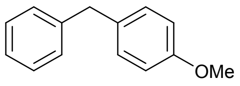

δ_H (400 MHz, $CDCl_3$) 7.31 – 7.22 (2 H, m), 7.20 – 7.14 (3 H, m), 7.10 (2 H, d, *J* 8.8), 6.82 (2 H, d, *J* 8.7), 3.92 (2 H, s), 3.77 (3 H, s); δ_C (101 MHz, $CDCl_3$) 158.1, 141.7, 133.4, 130.0, 128.9, 128.6, 126.1, 114.0, 55.4, 41.2.

1,4-dimethyl-2-(2-methylbenzyl)benzene (**2e**)[4]

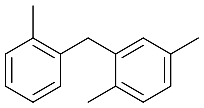

2.14 g (10.15 mmol, 50.8% yield) from 20 mmol scale experiment. δ $_H$ (400 MHz, $CDCl_3$) 7.18 (1 H, dd, *J* 7.3, 1.5), 7.16 – 7.04 (3 H, m), 6.96 (1 H, dd, *J* 7.6, 1.4), 6.86 (1 H, dd, *J* 7.5, 1.6), 6.73 (1 H, s), 3.87 (2 H, s), 2.28 (3 H, s), 2.24 (3 H, s), 2.21 (3 H, s); δ $_C$ (101 MHz, $CDCl_3$) 138.7, 138.3, 136.7, 135.6, 133.6, 130.2, 130.1, 130.1, 129.1, 127.0, 126.3, 126.1, 36.8, 21.2, 19.8, 19.2.

1,4-dimethyl-2-(3-methylbenzyl)benzene (**2f**)

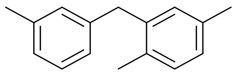

2.34 g (11.07 mmol, 55.4% yield) from 20 mmol scale experiment. δ $_H$ (400 MHz, $CDCl_3$) 7.15 (1 H, t, *J* 7.5), 7.04 (1 H, d, *J* 7.6), 7.01 – 6.86 (5 H, m), 3.91 (2 H, s), 2.30 (3 H, s), 2.28 (3 H, s), 2.20 (3 H, s); δ $_C$ (101 MHz, $CDCl_3$) 140.6, 138.9, 138.0, 135.5, 133.6, 130.9, 130.3, 129.6, 128.4, 127.2, 126.8, 125.9, 39.5, 21.6, 21.1, 19.4.

1,4-dimethyl-2-(4-methylbenzyl)benzene (**2g**)[4]

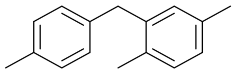

2.69 g (12.8 mmol, 64.0% yield) from 20 mmol scale experiment. δ $_H$ (400 MHz, $CDCl_3$) 7.14 – 6.98 (5 H, m), 6.97 – 6.91 (2 H, m), 3.90 (2 H, s), 2.31 (3 H, s), 2.28 (3 H, s), 2.19 (3 H, s); δ $_C$ (101 MHz, $CDCl_3$) 139.1, 137.6, 135.5, 135.4, 133.5, 130.8, 130.3, 129.2, 128.7, 127.1, 39.1, 21.1, 21.1, 19.3.

1-(4-chlorobenzyl)-2,5-dimethylbenzene (**2i**)[3]

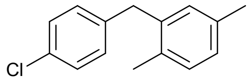

4.29 g (18.58 mmol, 92.9% yield) from 20 mmol scale experiment, chromatography; 91.2 g (395 mmol, 94%) from 422 mmol scale experiment, distillation (b.p. 124 °C, 0.75 mmHg). δ $_H$ (400 MHz, $CDCl_3$) 7.22 (2 H, d, *J* 8.5), 7.08 – 7.01 (3 H, m), 6.97 (1 H, d, *J* 7.6), 6.89 (1 H, s), 3.90 (2 H, s), 2.29 (3 H, s), 2.17 (3 H, s); δ $_C$ (101 MHz, $CDCl_3$) 139.2, 138.3, 135.7, 131.8, 130.8, 130.5, 130.2, 128.6, 127.5, 38.9, 21.1, 19.3.

1-cyclohexyl-2,5-dimethylbenzene (**2j**)[5]

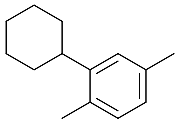

2.71 g (14.39 mmol, 71.9% yield) from 20 mmol scale experiment. GC conversion was not determined due to peak overlap of cyclohexanol and *p*-xylene. δ $_H$ (400 MHz, $CDCl_3$) 7.05 – 6.98 (2 H, m), 6.89 (1 H, d, *J* 7.8), 2.67 (1 H, tt, *J* 11.5, 3.0), 2.30 (3 H, s), 2.28 (3 H, s), 1.94 – 1.67 (5 H, m), 1.48 – 1.20 (5 H,

m); δ C (101 MHz, $CDCl_3$) 145.9, 135.5, 132.1, 130.2, 126.3, 126.3, 33.8, 27.4, 26.5, 21.3, 19.0.

1-(1-Adamantyl)-2,5-dimethylbenzene (**2k**)

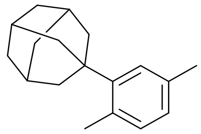

0.61 g (2.55 mmol, 25.5% yield) from 10 mmol scale experiment. δ H (400 MHz, $CDCl_3$) 7.14 (1 H, d, *J* 1.2), 7.01 (1 H, d, *J* 7.6), 6.92 (1 H, dd, *J* 7.6, 1.2), 2.58 (3 H, s), 2.31 (3 H, s), 2.09 (9 H, s), 1.79 (6 H, s); δ C (101 MHz, $CDCl_3$) 147.8, 135.1, 133.2, 133.1, 127.0, 126.4, 41.5, 37.9, 37.1, 29.3, 23.1, 21.4; IR (neat, ATR-diamond) 2967, 2889, 2847, 1491, 1448, 1342, 1168, 1097, 1032, 973, 875, 809, 724, 645, 582 (cm^{-1}); HRMS (EI+): m/z calcd. for $C_{18}H_{24}$, 240.1878. found 240.1880.

References

1 B. Török, I. Kiricsi, Á. Molnár and G. A. Olah, *J. Catal.*, 2000, **193**, 132–138.

2 M. Ohsumi and R. Kuwano, *Chem. Lett.*, 2008, **37**, 796–797.

3 N. Sakai, K. Kawana, R. Ikeda, Y. Nakaike and T. Konakahara, *European J. Org. Chem.*, 2011, 3178–3183.

4 F. Wang and W. Ueda, *Chem. - A Eur. J.*, 2009, **15**, 742–753.

5 Y. Yamamoto and K. Itonaga, *Chem. - A Eur. J.*, 2008, **14**, 10705–10715.

Reaction profiles

Raw reaction profiles (conversion/yield – time plot) of experiments were summarized in this section.

Table 1

Entry 1. Zeolite H-Y

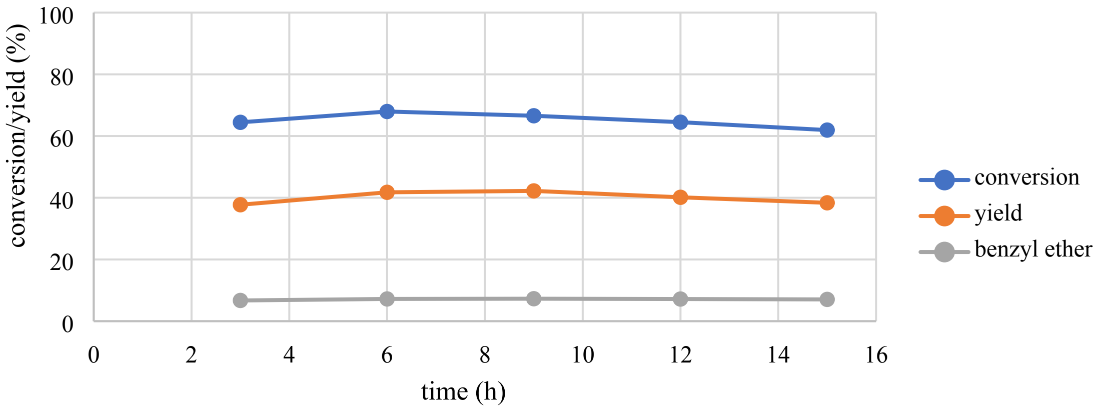

Entry 2. Zeolite H-beta

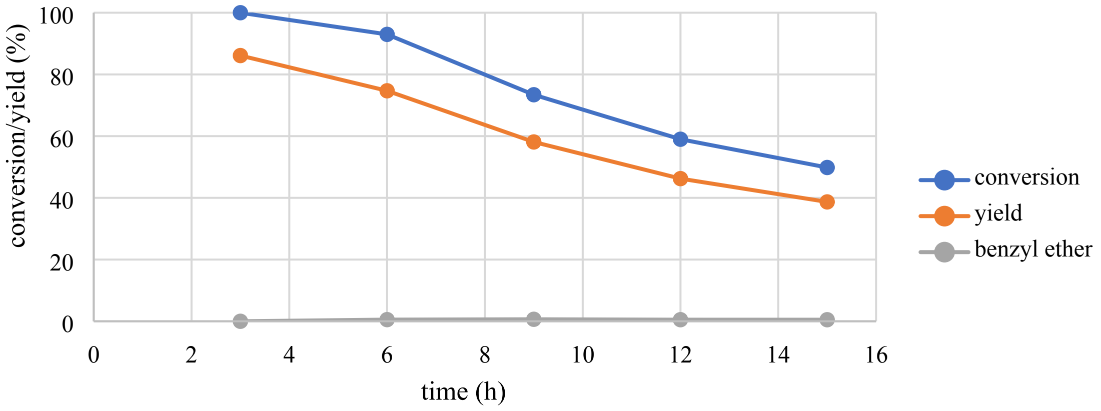

Entry 3. Nafion-Silica

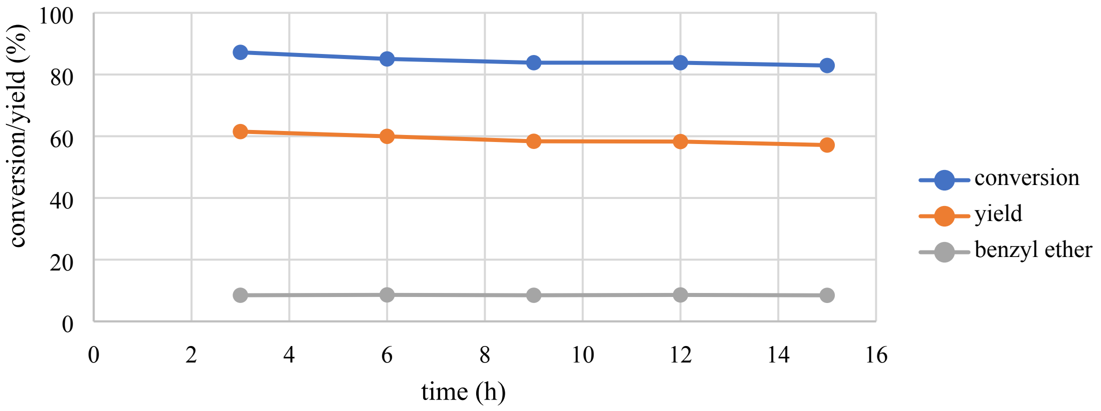

Entry 4. Sulphated ZrO_2

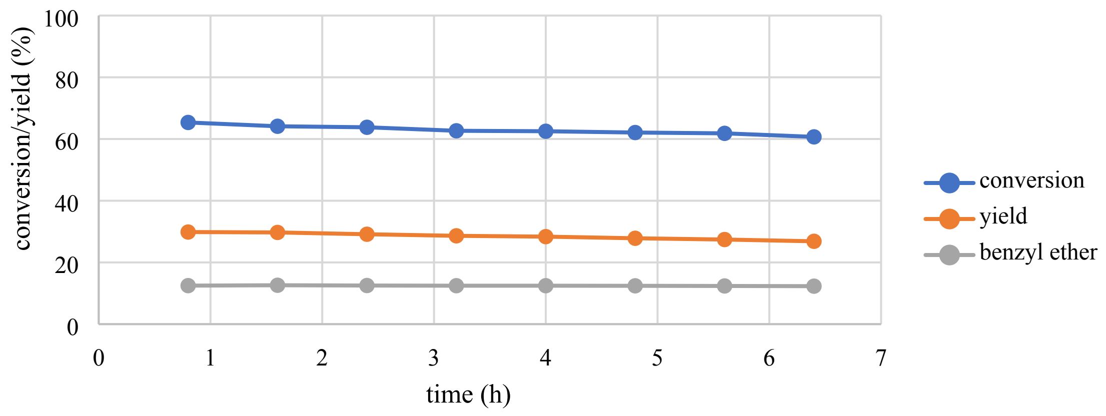

Entry 5. Montmorillonite K10

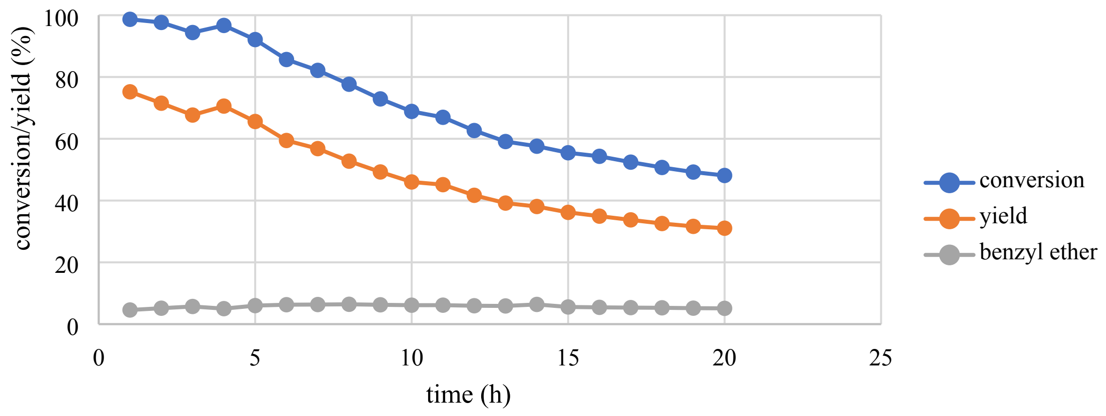

Entry 6. Bentonite

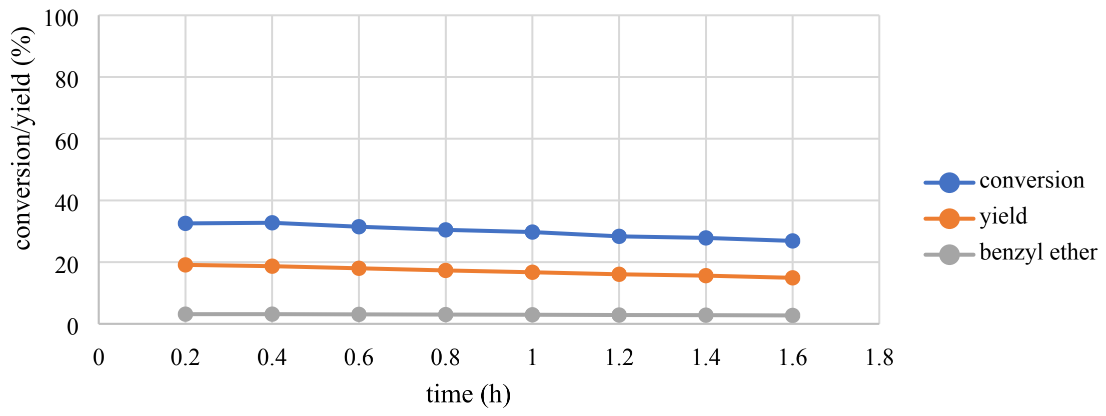

Entry 7. H-Mont

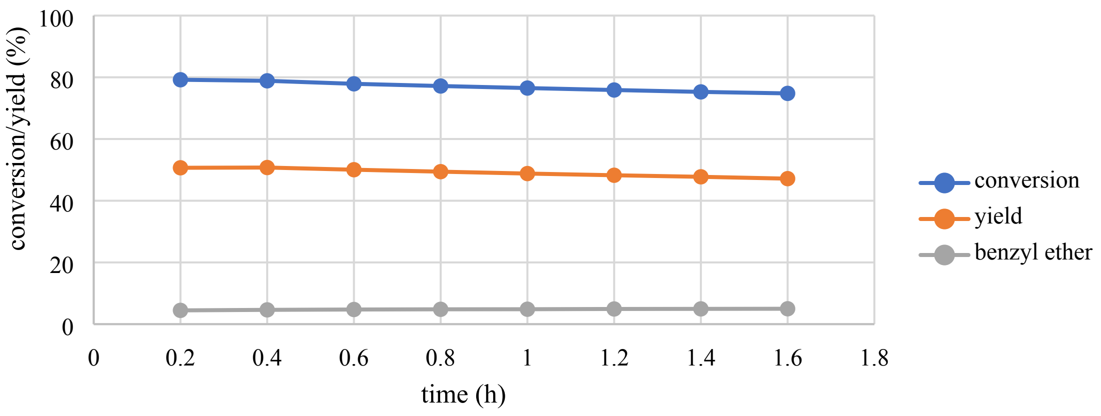

Table 2

Entry 1. HCl (1 M) treatment, 1/9 (catalyst: 16.4 mg)

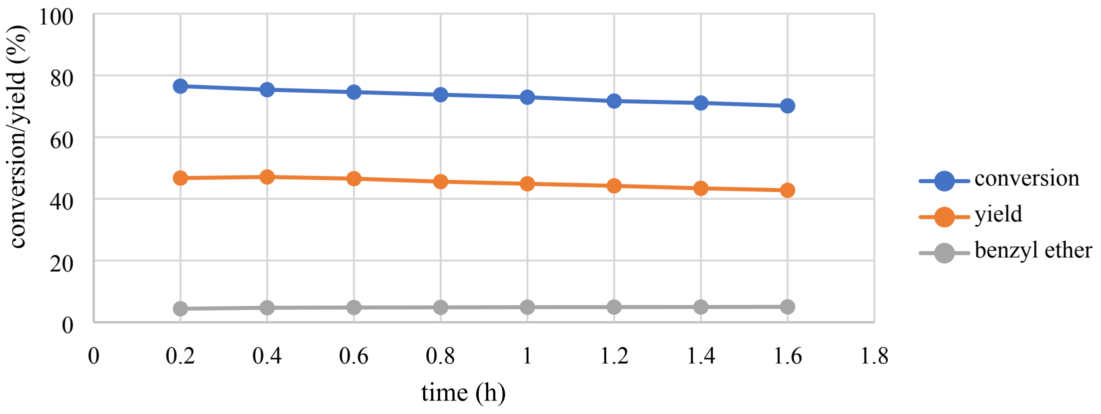

Entry 2. HCl (conc.) treatment, 1/9 (catalyst: 16.1 mg)

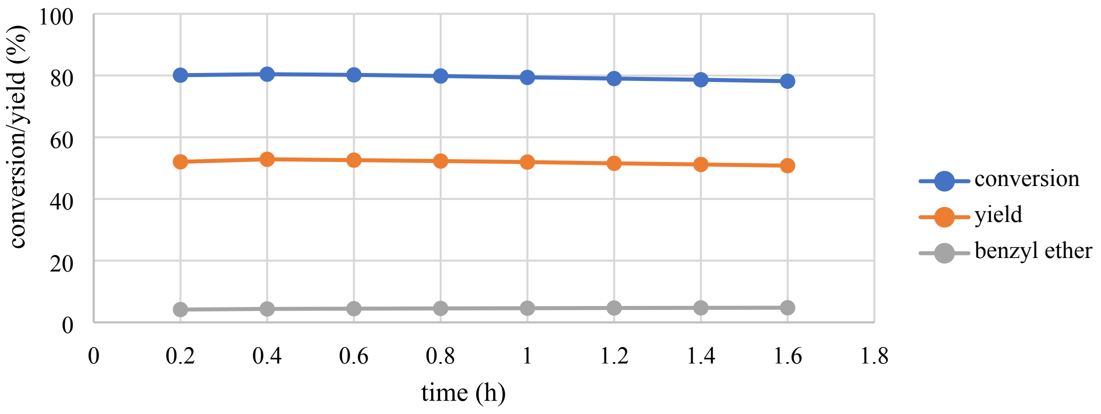

Entry 3. HCl (1 M) treatment, 1/99 (catalyst: 1.92 mg)

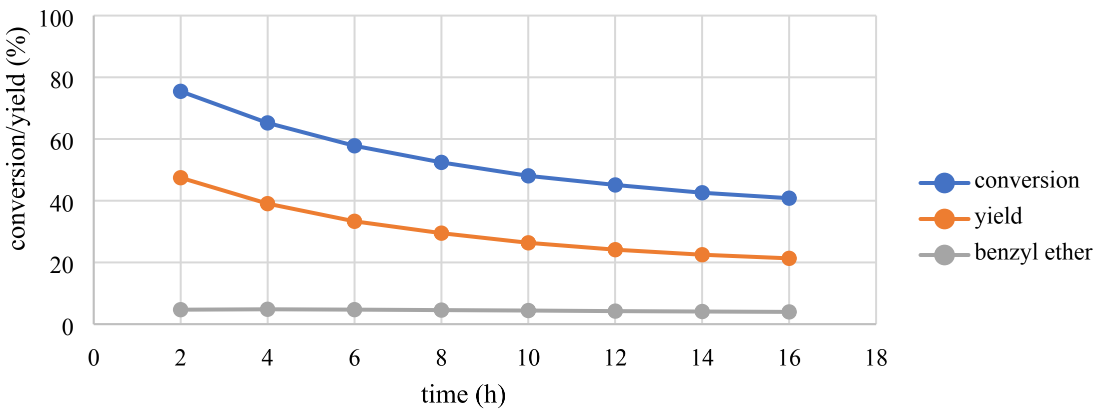

Entry 4. HCl (conc.) treatment, 1/99 (catalyst: 3.36 mg)

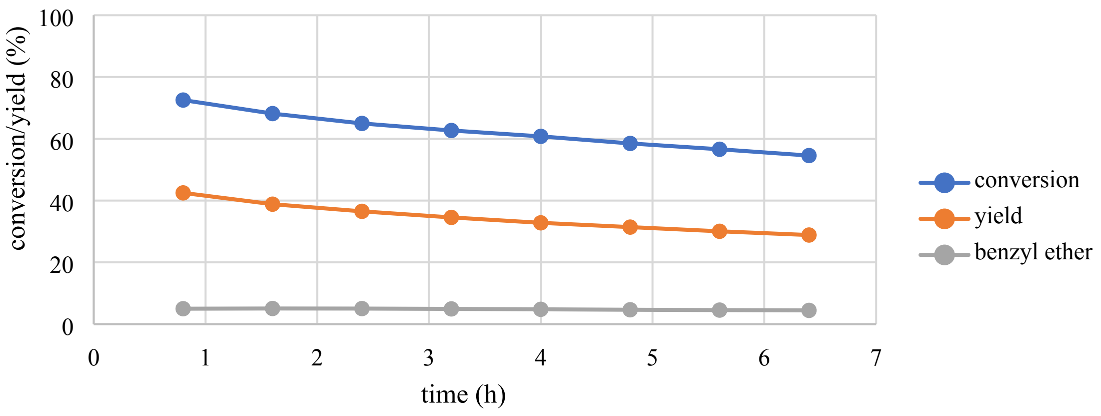

Substrate scope (scheme 2)

1-benzyl-2,5-dimethylbenzene (**2a**) (catalyst: 47.7 mg)

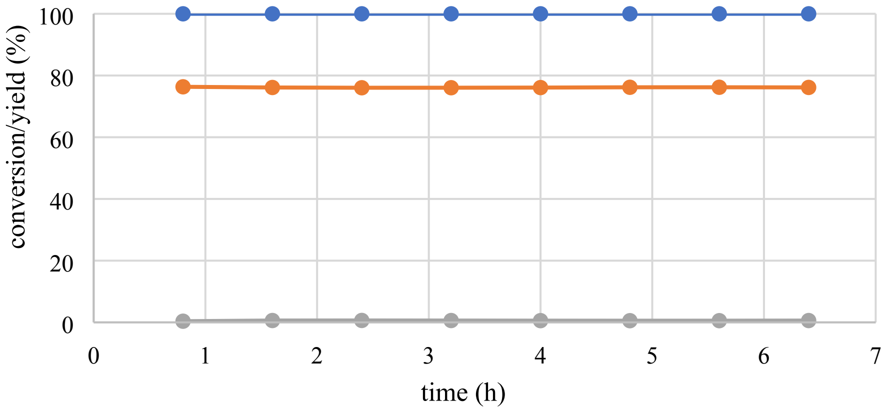

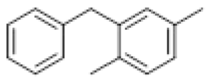

o-benzyltoluene and *p*-benzyltoluene (**2c**), obtained as an inseparable mixture. GC trace describes a combined yield of o- and *p*- products, calculated from sum of their peak areas. (catalyst: 85.3 mg)

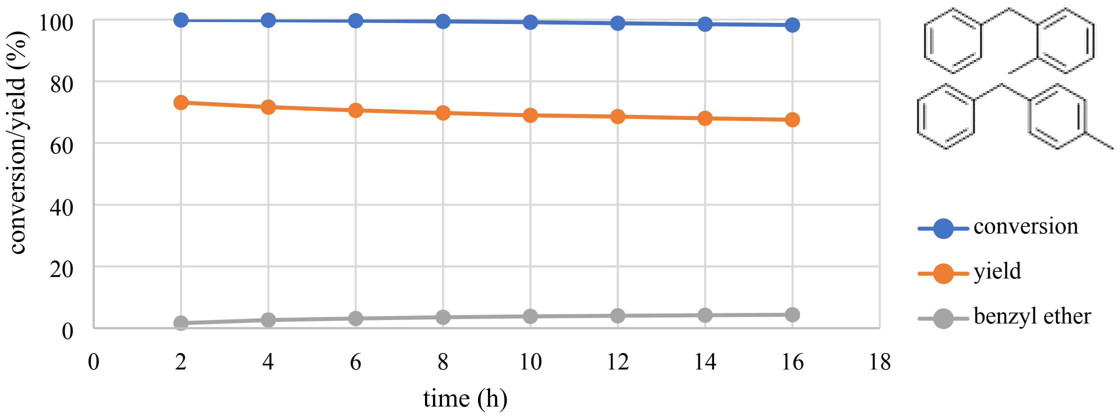

o-benzylanisole and *p*-benzylanisole (**2d**) (catalyst: 50.7 mg)

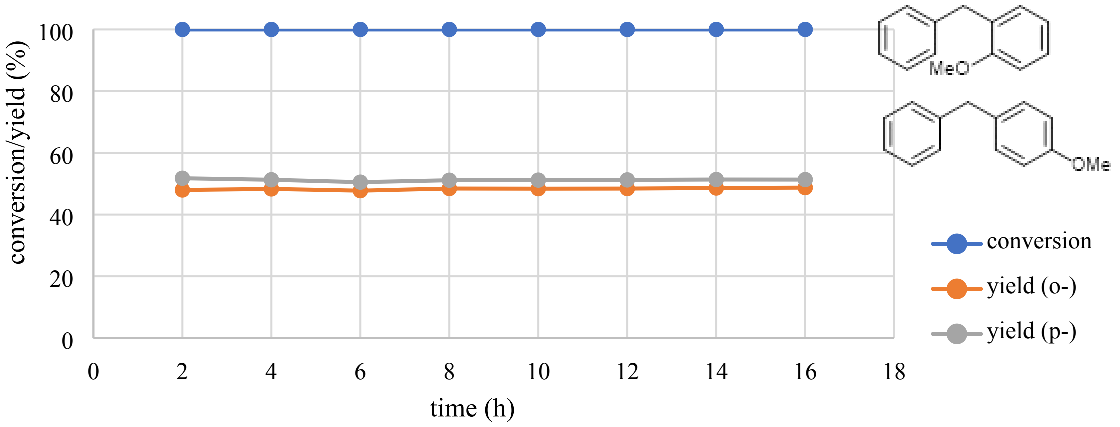

1,4-dimethyl-2-(2-methylbenzyl)benzene (**2e**) (catalyst: 46.1 mg)

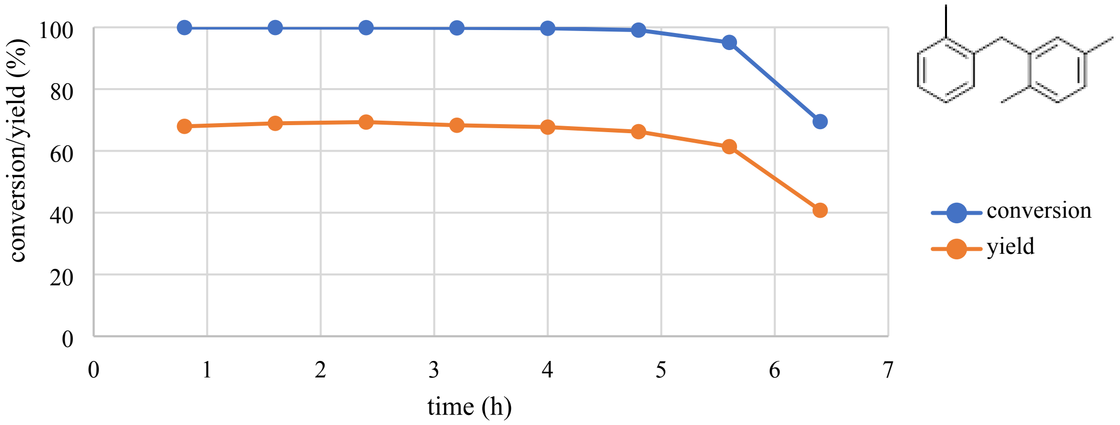

1,4-dimethyl-2-(3-methylbenzyl)benzene (**2f**) (catalyst: 41.9 mg)

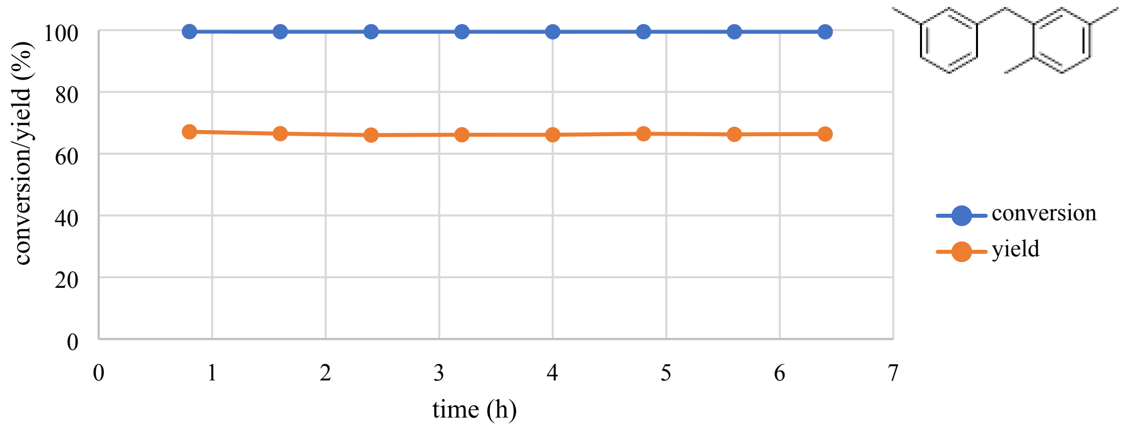

1,4-dimethyl-2-(4-methylbenzyl)benzene (**2g**) (catalyst: 46.1 mg)

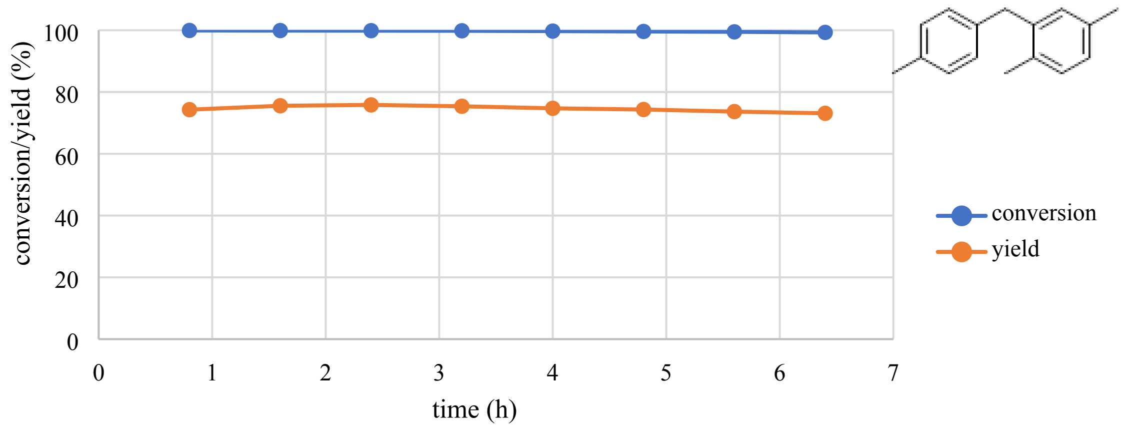

1-(4-chlorobenzyl)-2,5-dimethylbenzene (**2i**) (catalyst: 47.3 mg)

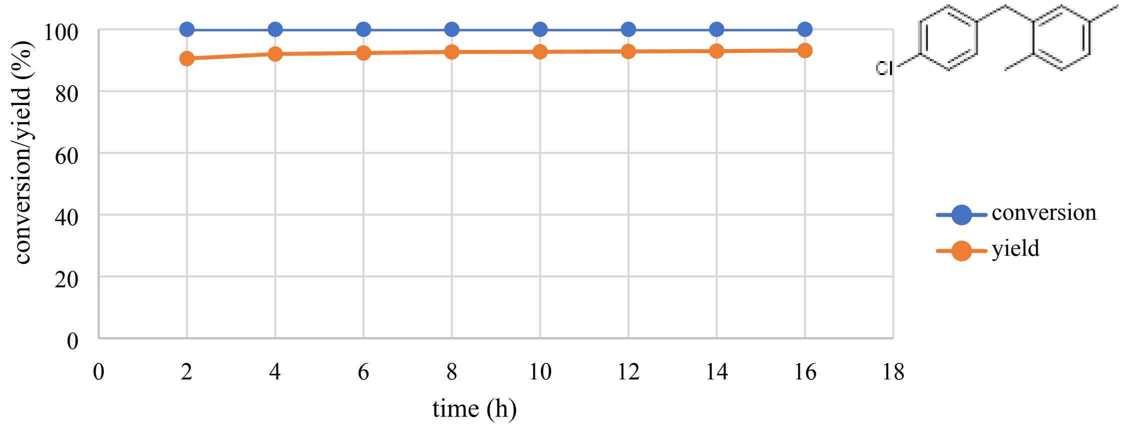

1-cyclohexyl-2,5-dimethylbenzene (**2j**). Conversion was not recorded due to peak overlap of cyclohexanol with *p*-xylene. (catalyst: 181.9 mg)

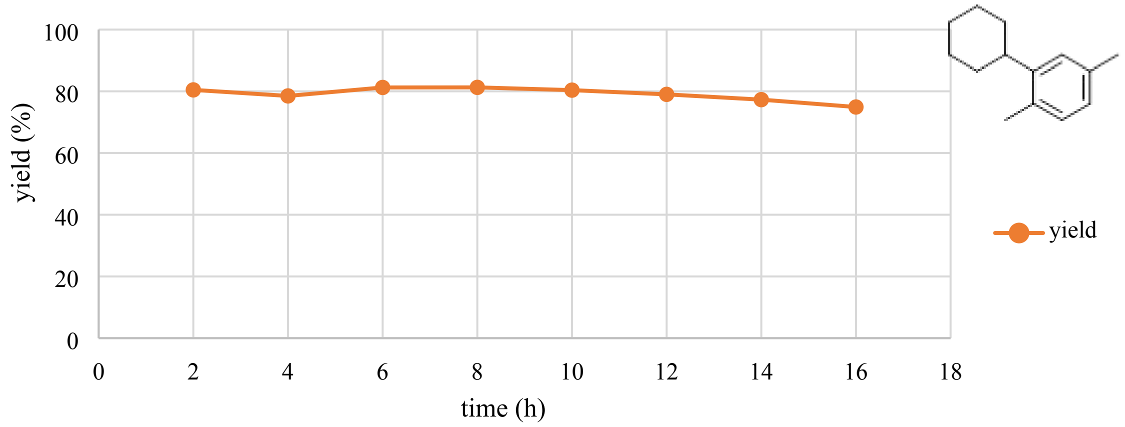

1-(1-Adamantyl)-2,5-dimethylbenzene (**2k**) (catalyst: 387.8 mg)

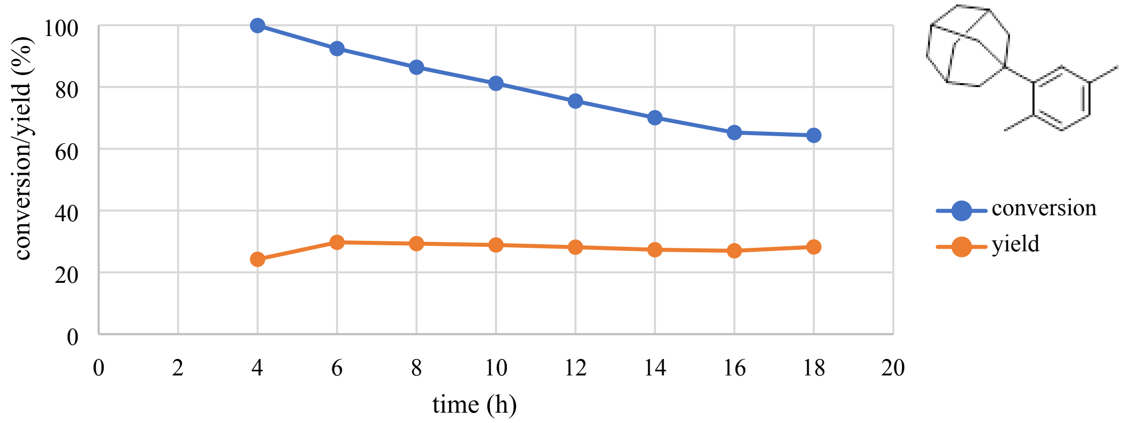

NMR spectra

1-benzyl-2,5-dimethylbenzene (**2a**)

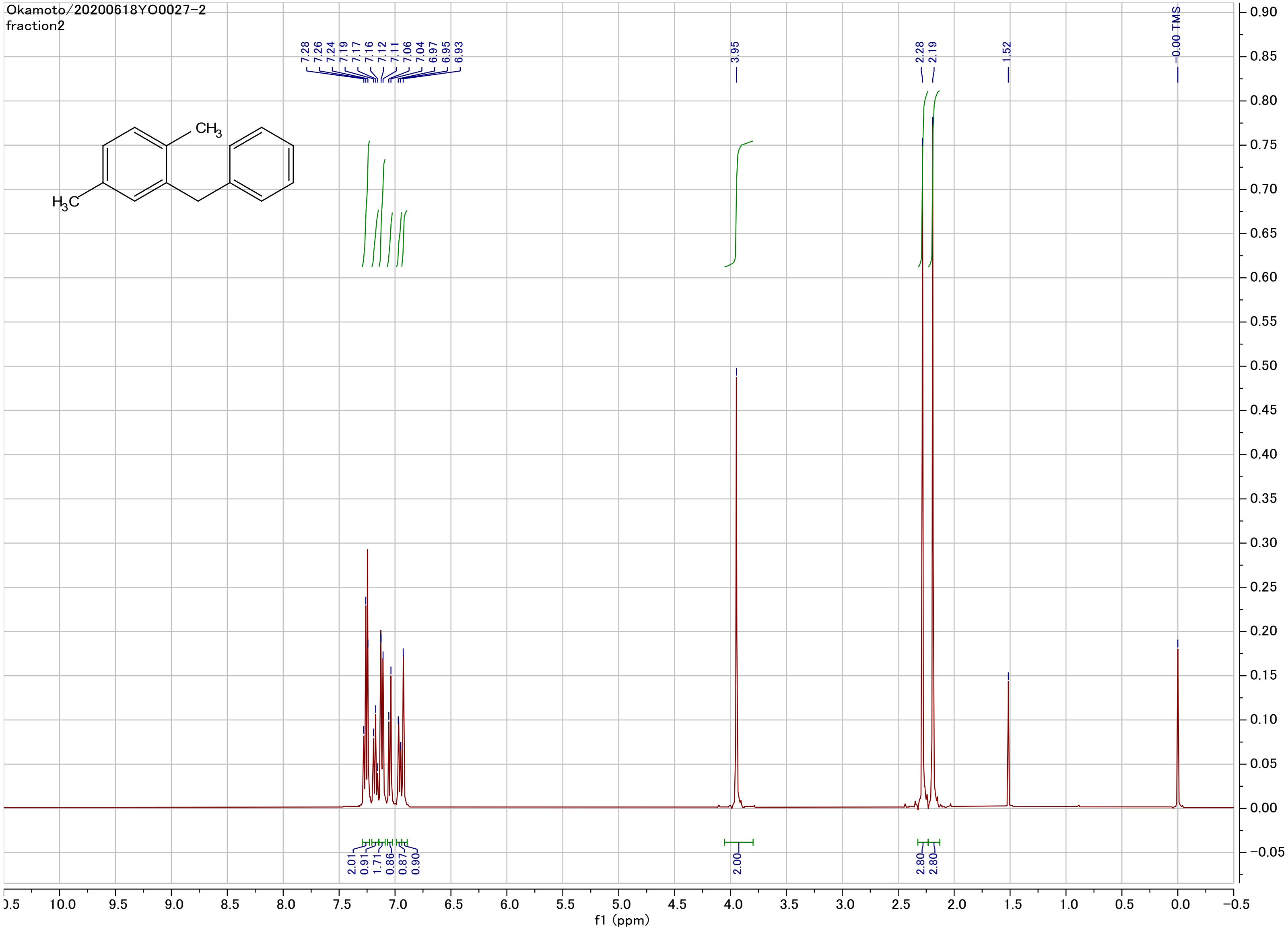

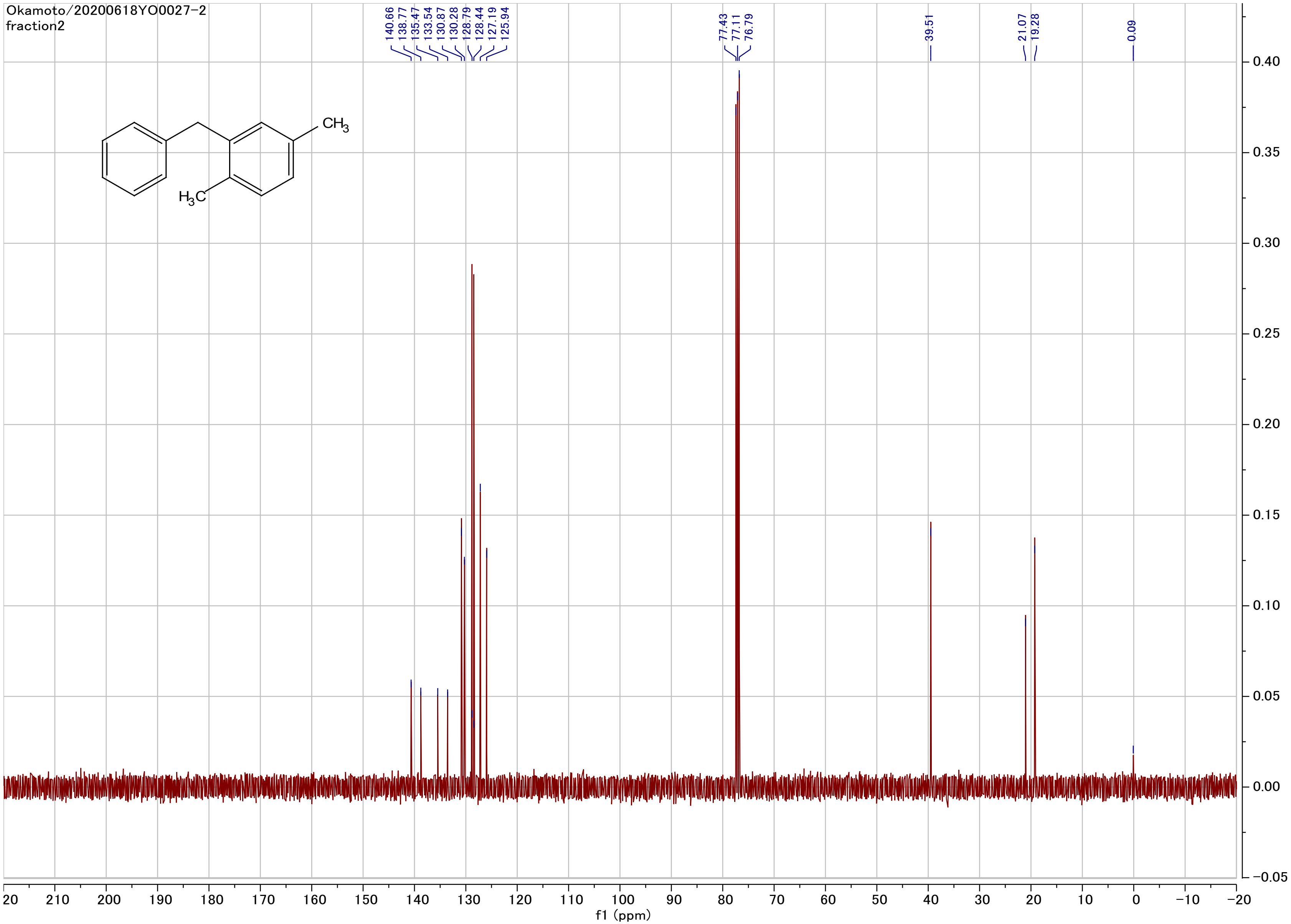

Okamoto/20200618YO0027-2
fraction2
140.66
138.77
135.47
133.54
130.87
130.28
128.79
128.44
127.19
125.94
77.43
77.11
76.79
39.51
21.07
19.28
0.09
CH3
H3C
f1 (ppm)

o-benzyltoluene and *p*-benzyltoluene (**2b**), obtained as an inseparable mixture

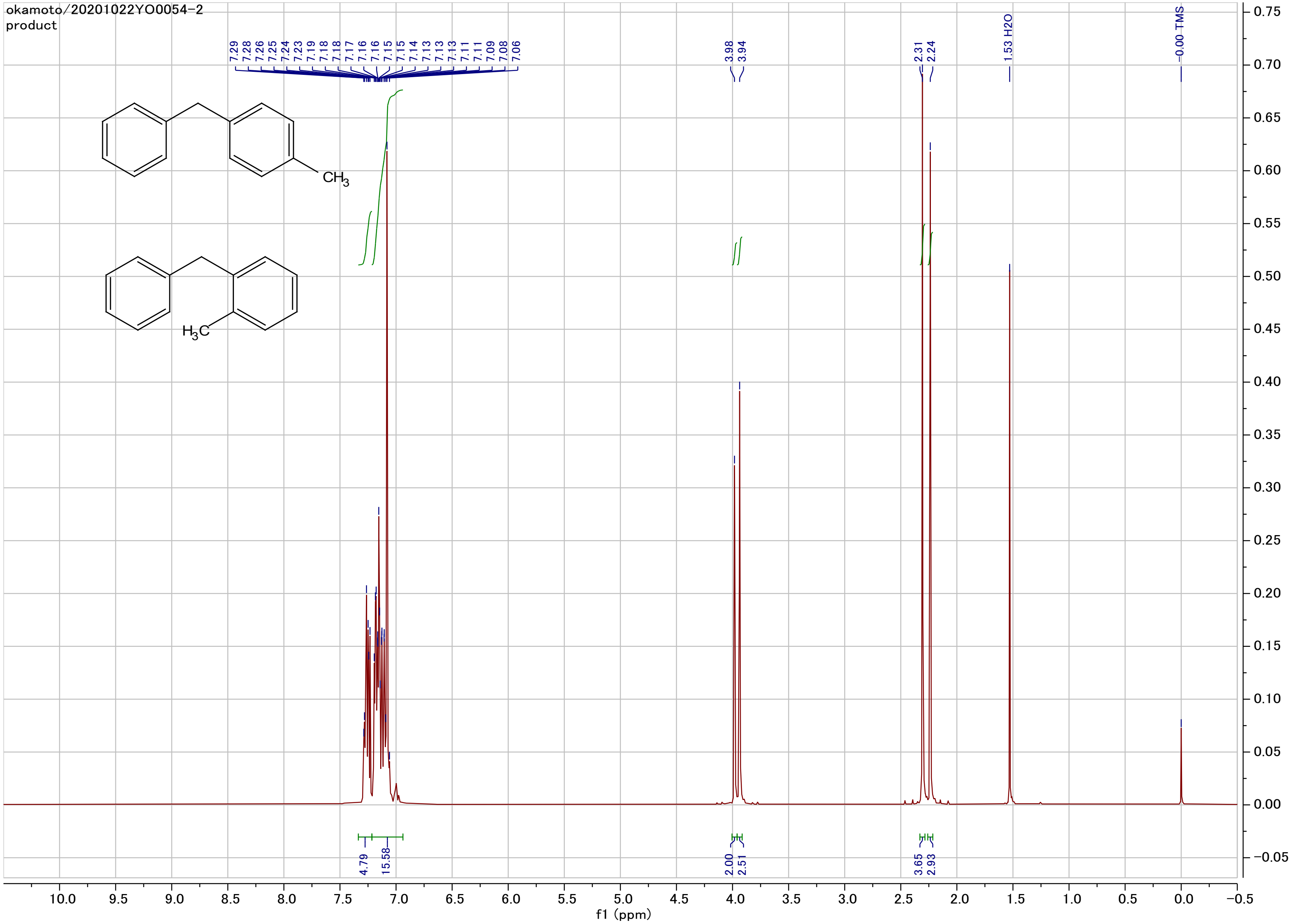

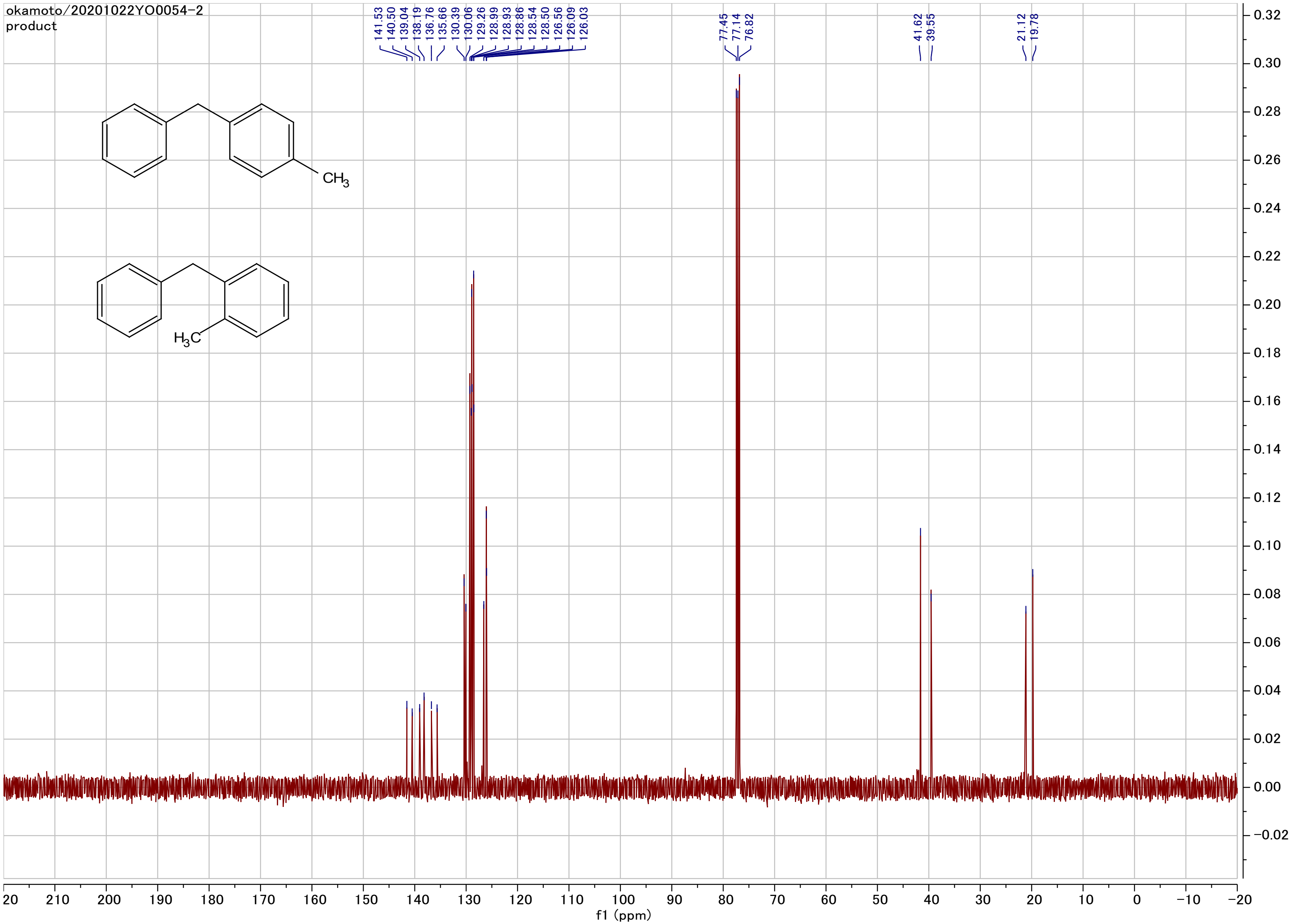
okamoto/20201022YO0054-2
product
141.53
140.50
139.04
138.19
136.76
135.66
130.39
130.06
129.26
128.99
128.93
128.86
128.54
128.50
126.56
126.09
126.03
77.45
77.14
76.82
41.62
39.55
21.12
19.78
CH3
H3C
f1 (ppm)

o-benzylanisole (**2d**)

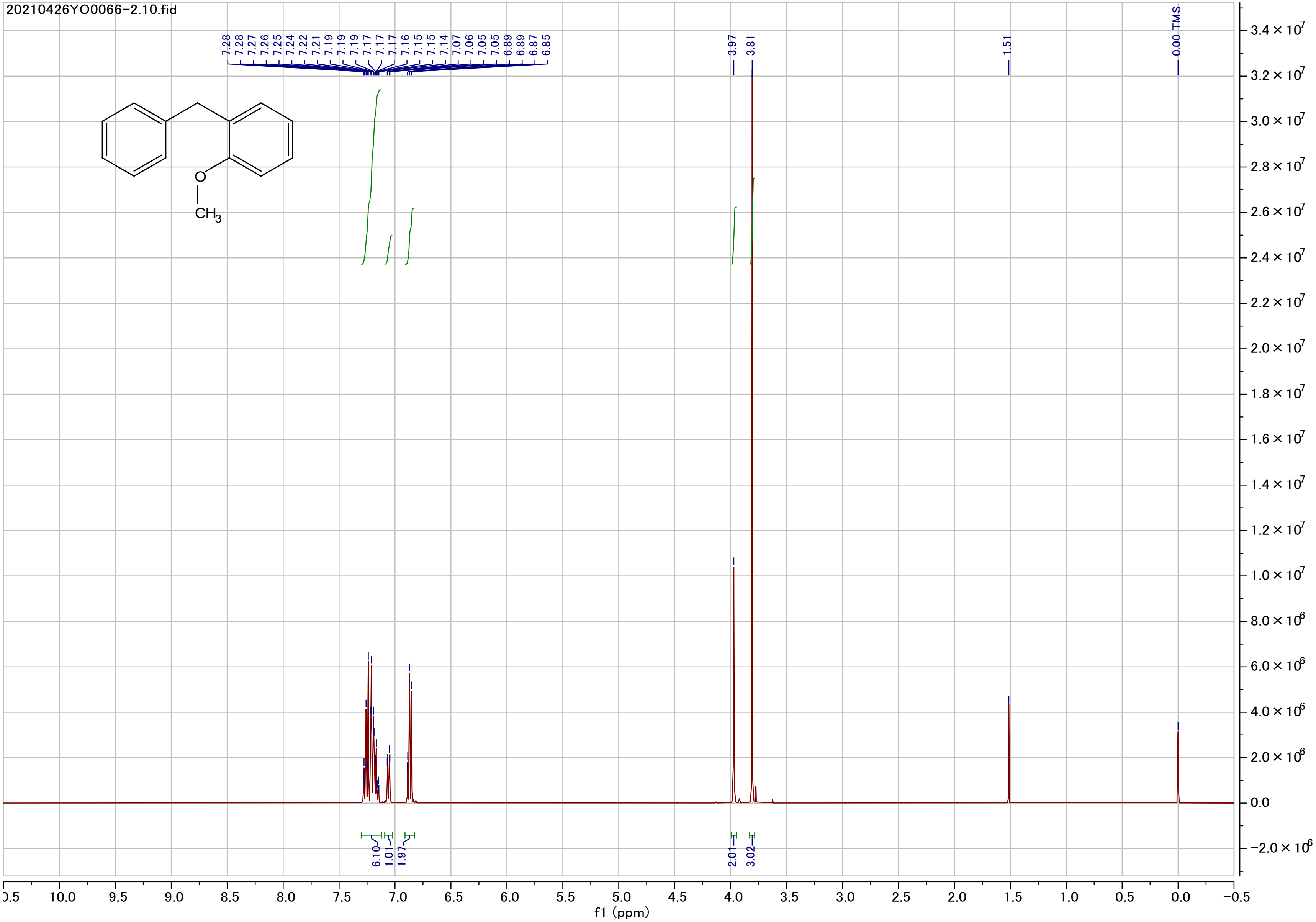

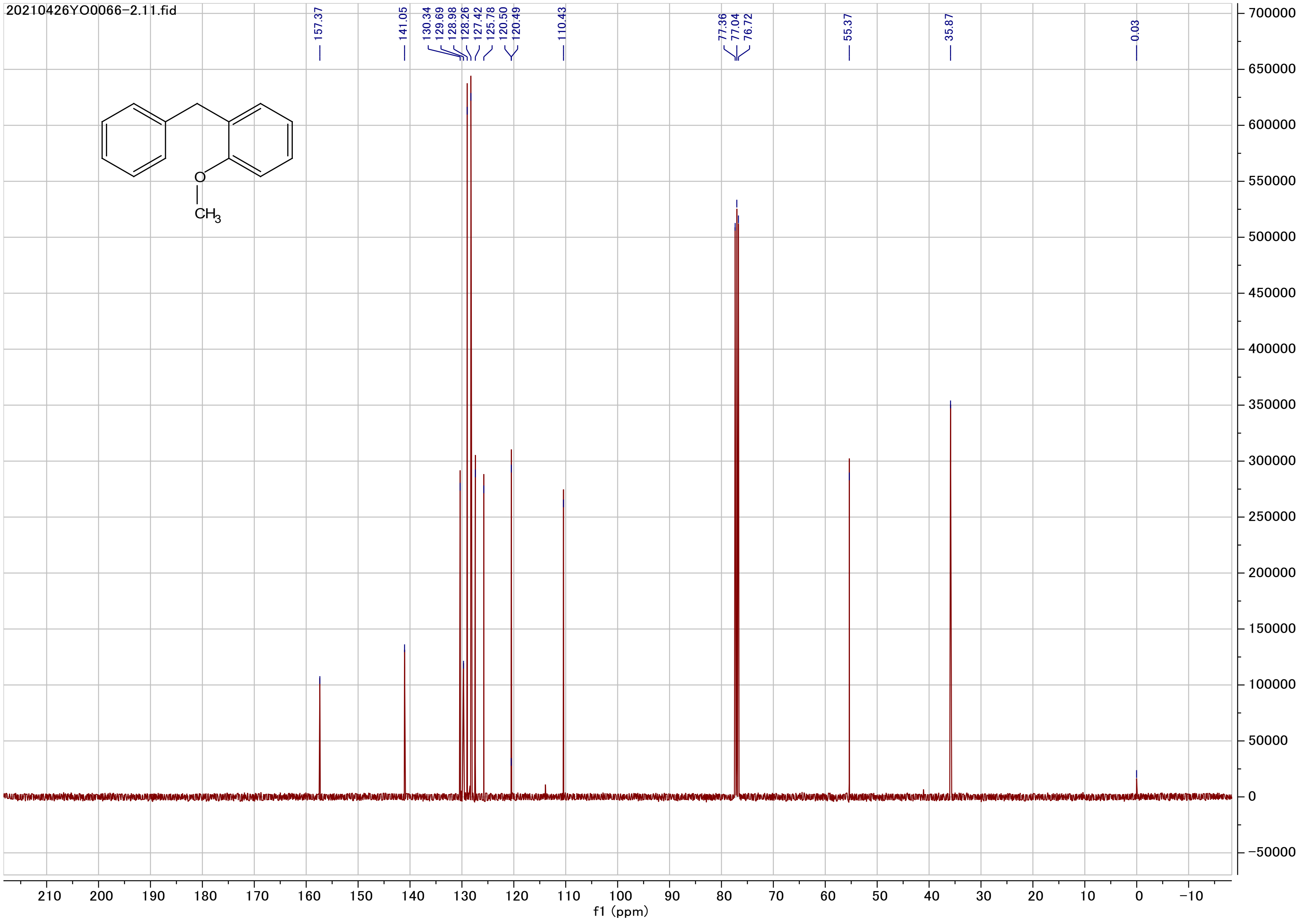
20210426YO0066-2.11.fid
157.37
141.05
130.34
129.69
128.98
128.26
127.42
125.78
120.50
120.49
110.43
77.36
77.04
76.72
55.37
35.87
0.03
O
CH3
f1 (ppm)

p-benzylanisole (**2d**)

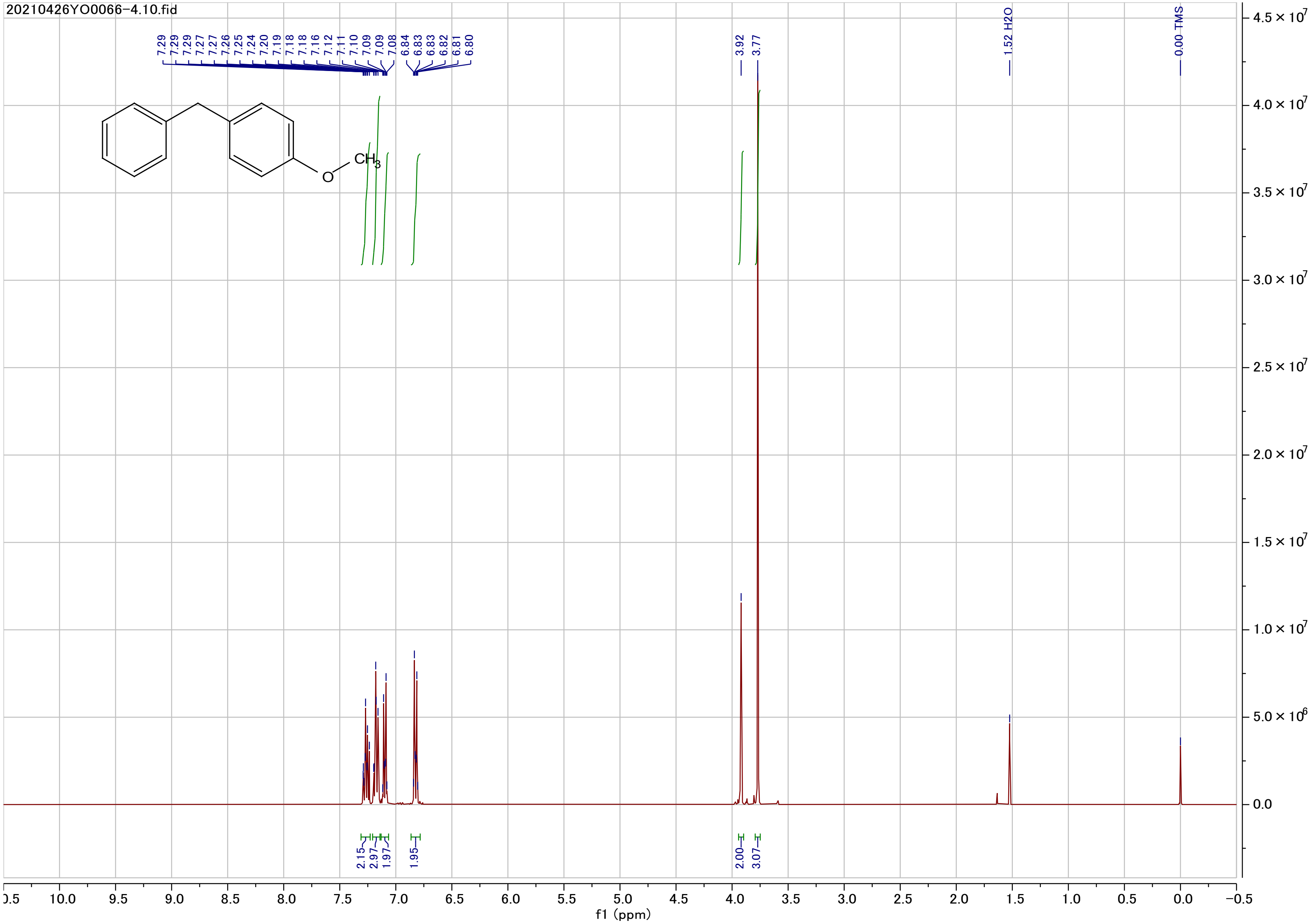

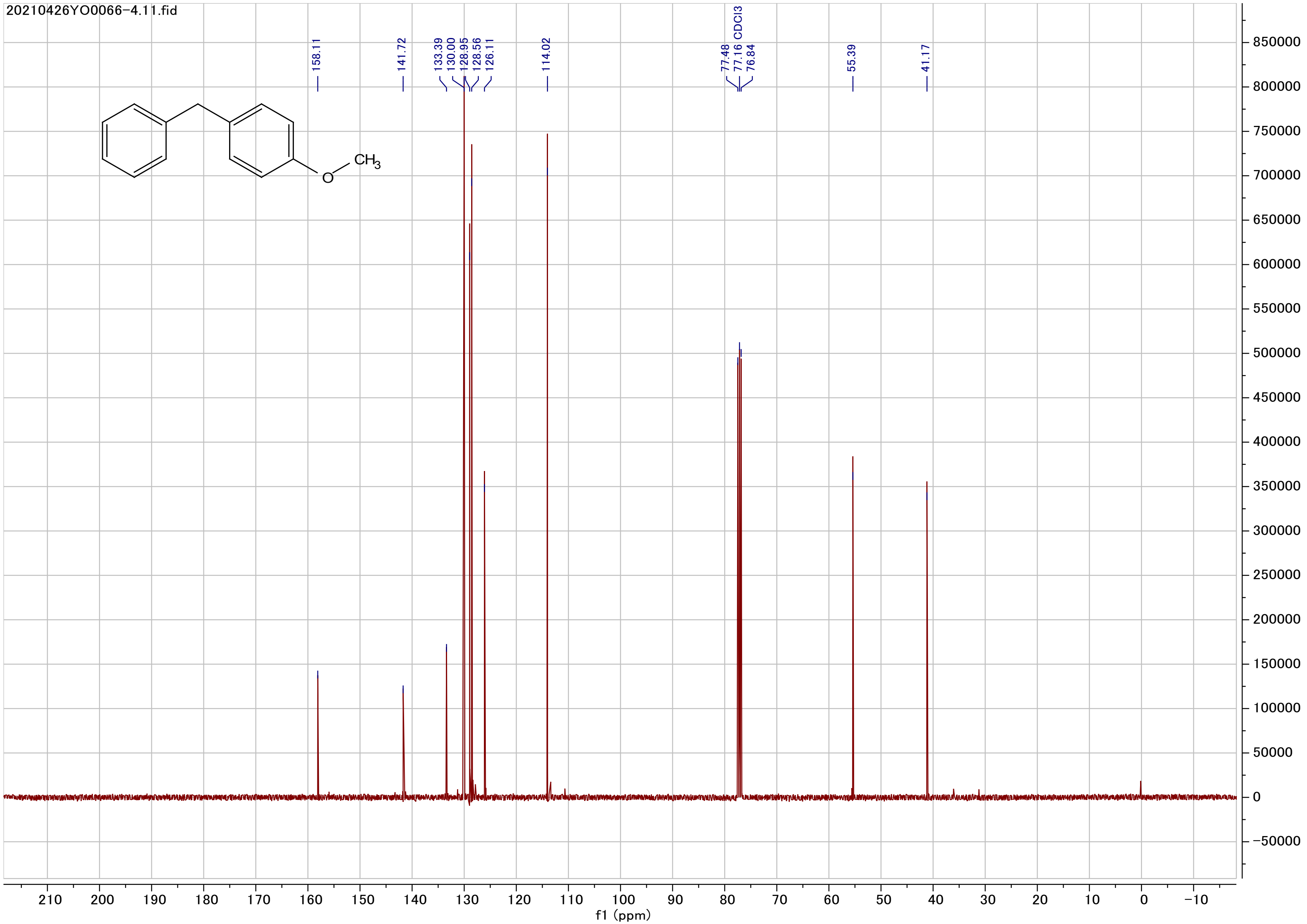
20210426YO0066-4.11.fid
158.11
141.72
133.39
130.00
128.95
128.56
126.11
114.02
77.48
77.16 CDCl3
76.84
55.39
41.17
O
CH3
f1 (ppm)

1,4-dimethyl-2-(2-methylbenzyl)benzene (**2e**)

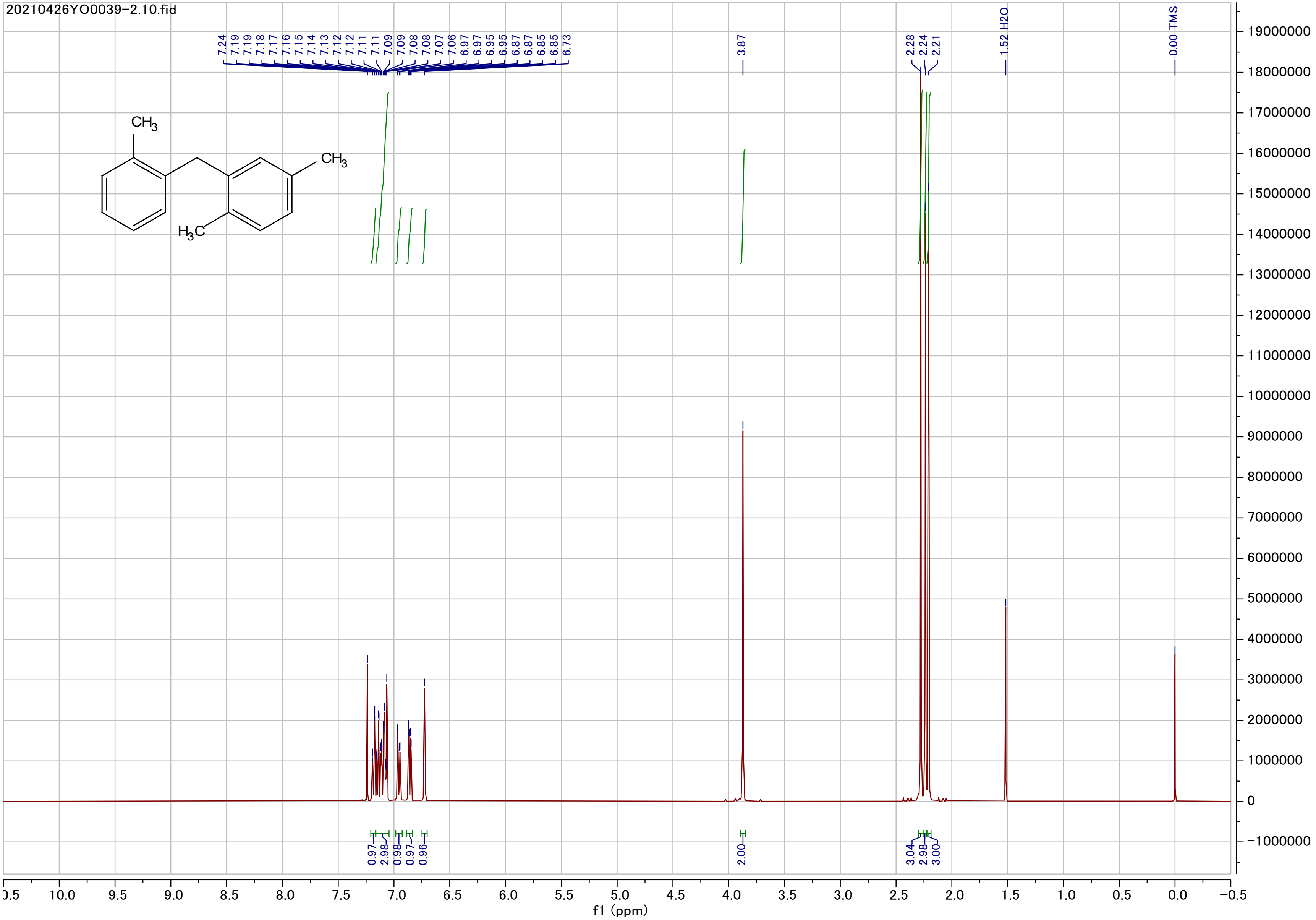

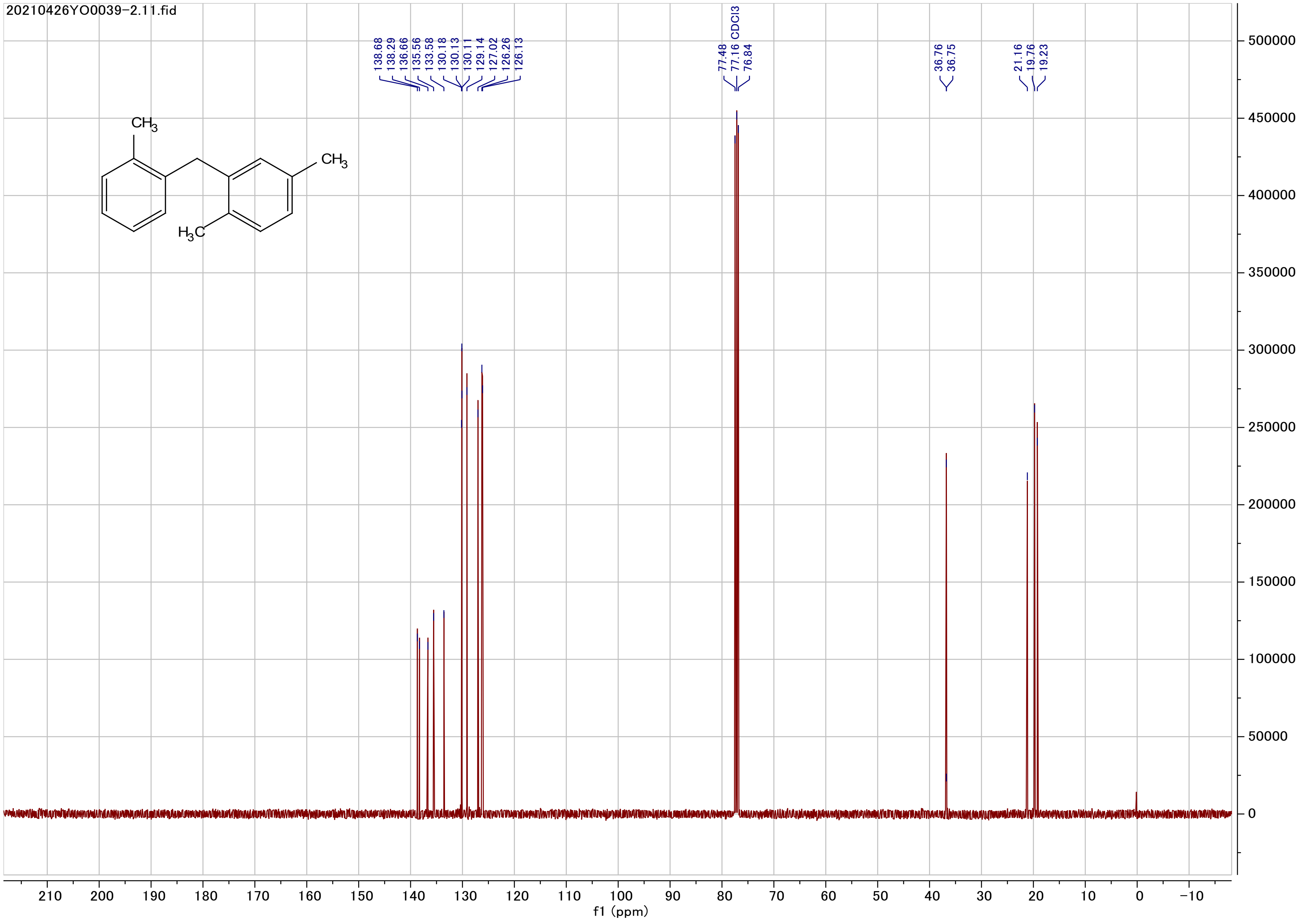
20210426YO0039-2.11.fid
138.68
138.29
136.66
135.56
133.58
130.18
130.13
130.11
129.14
127.02
126.26
126.13
77.48
77.16 CDCl3
76.84
36.76
36.75
21.16
19.76
19.23
CH3
CH3
H3C
f1 (ppm)

1,4-dimethyl-2-(3-methylbenzyl)benzene (**2f**)

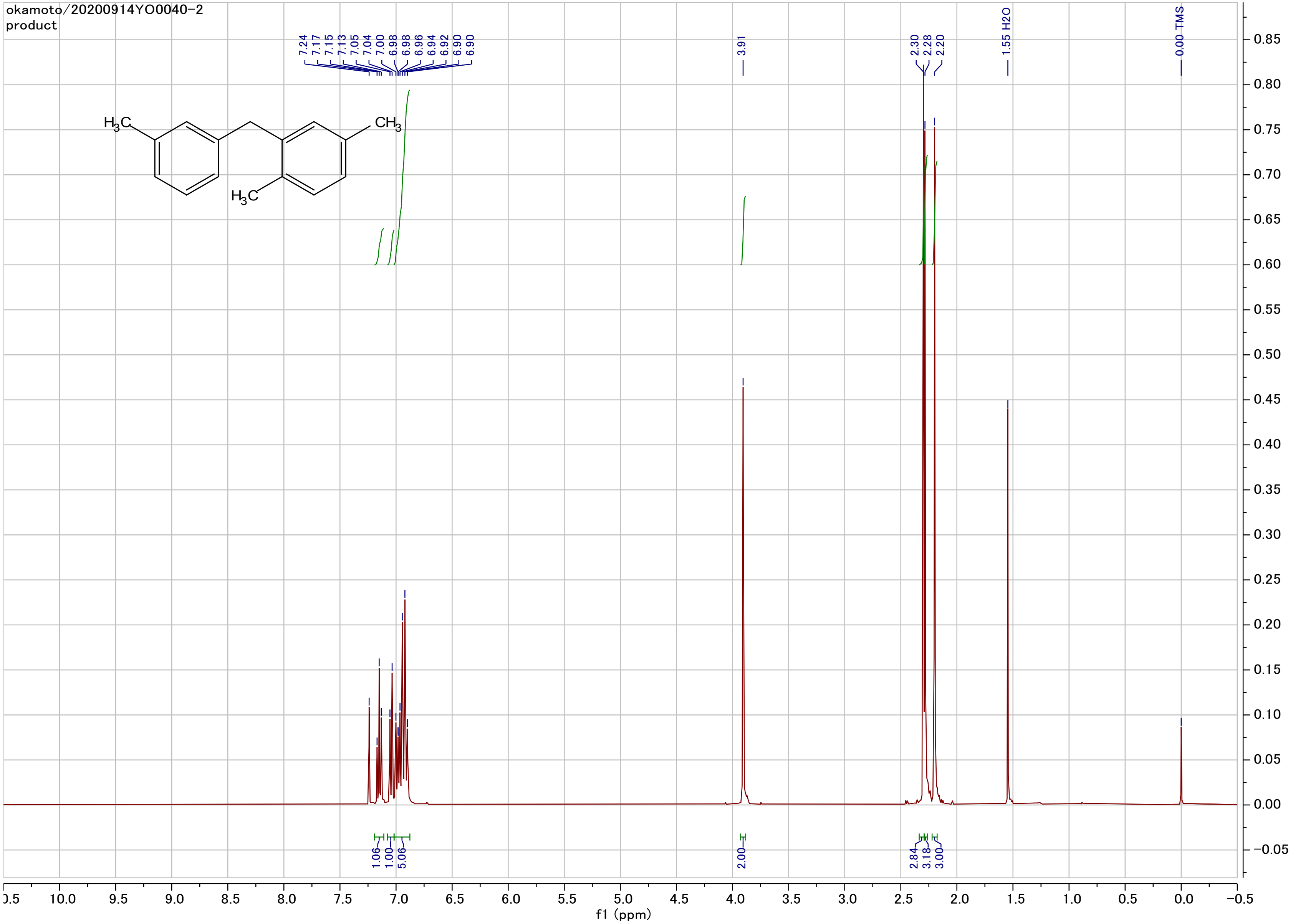

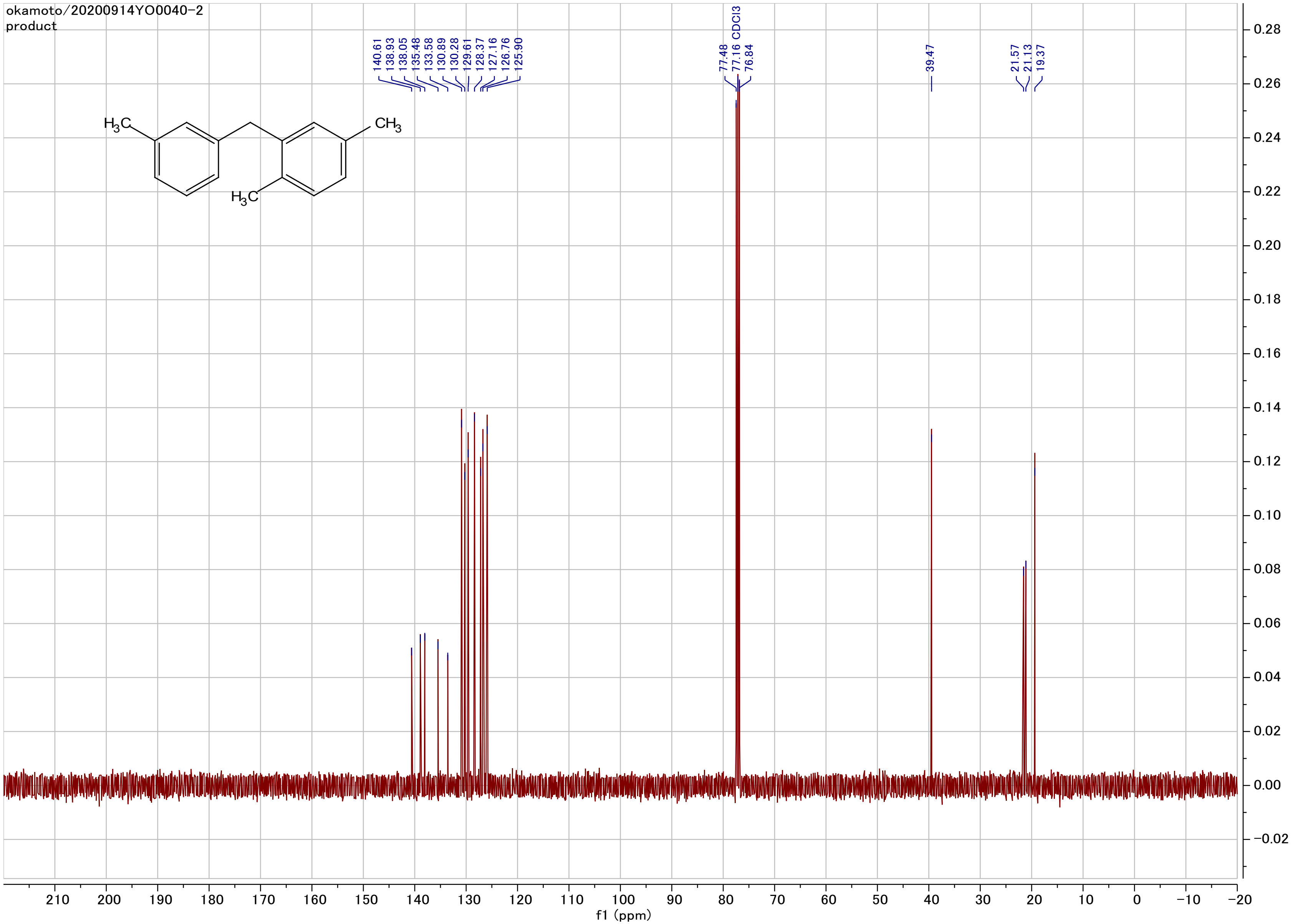
okamoto/20200914YO0040-2
product
140.61
138.93
138.05
135.48
133.58
130.89
130.28
129.61
128.37
127.16
126.76
125.90
77.48
77.16 CDCl3
76.84
39.47
21.57
21.13
19.37
H_3C
CH_3
H_3C
f1 (ppm)

1,4-dimethyl-2-(4-methylbenzyl)benzene (**2g**)

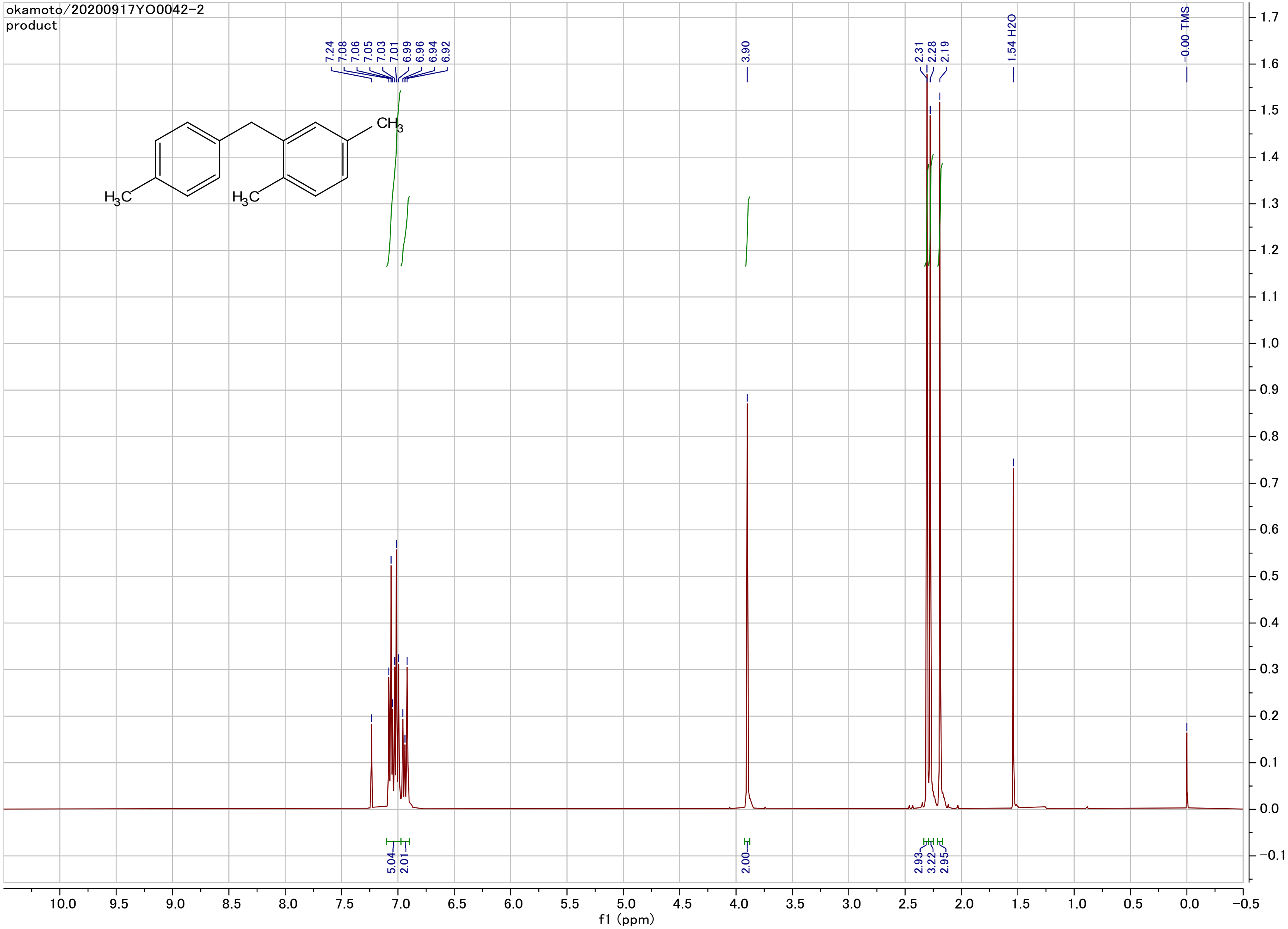

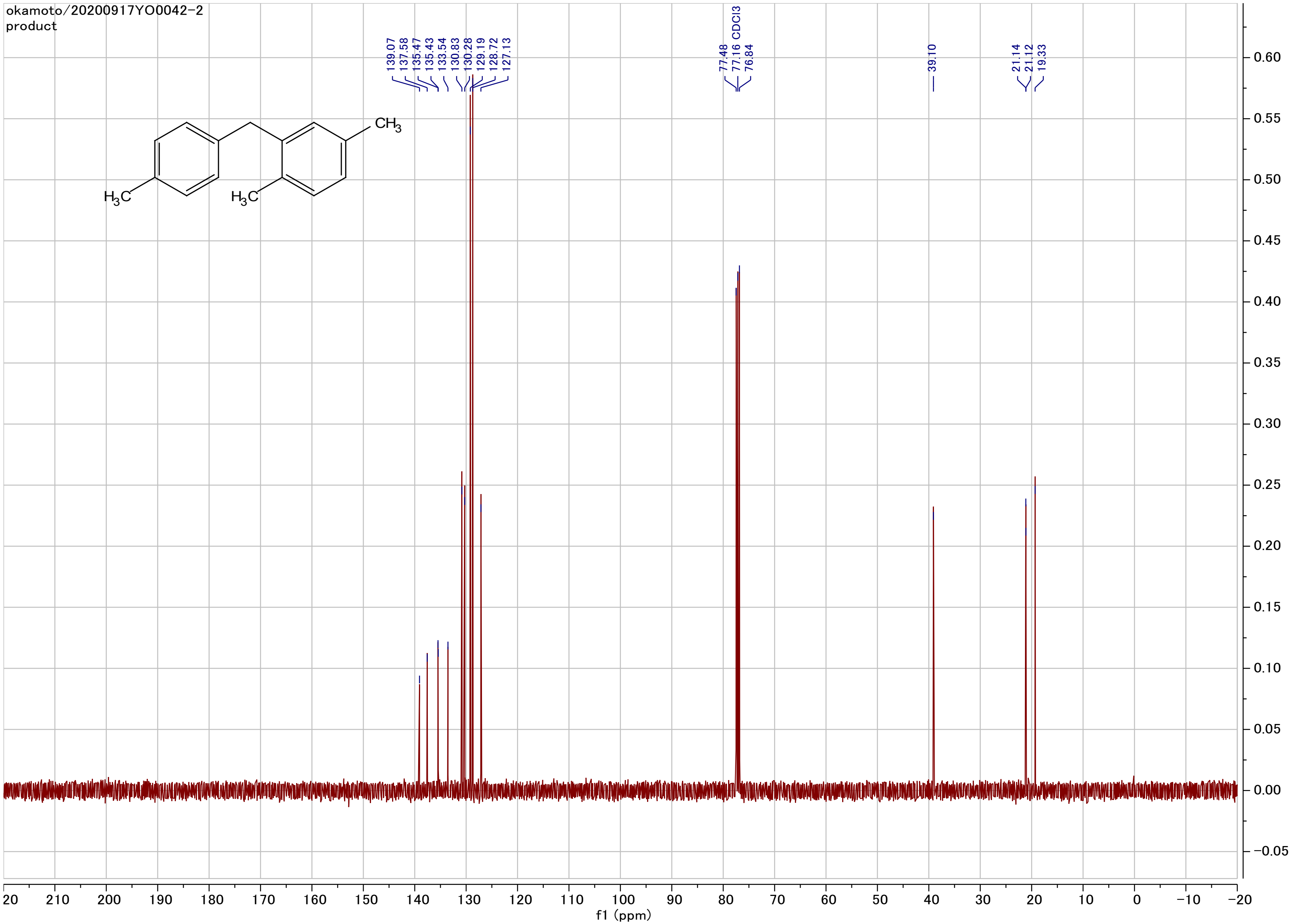
okamoto/20200917YO0042-2
product
139.07
137.58
135.47
135.43
133.54
130.83
130.28
129.19
128.72
127.13
77.48
77.16 CDCl3
76.84
39.10
21.14
21.12
19.33
CH3
H3C
H3C
f1 (ppm)

2-(4-chlorobenzyl)-1,4-dimethylbenzene (**2i**)

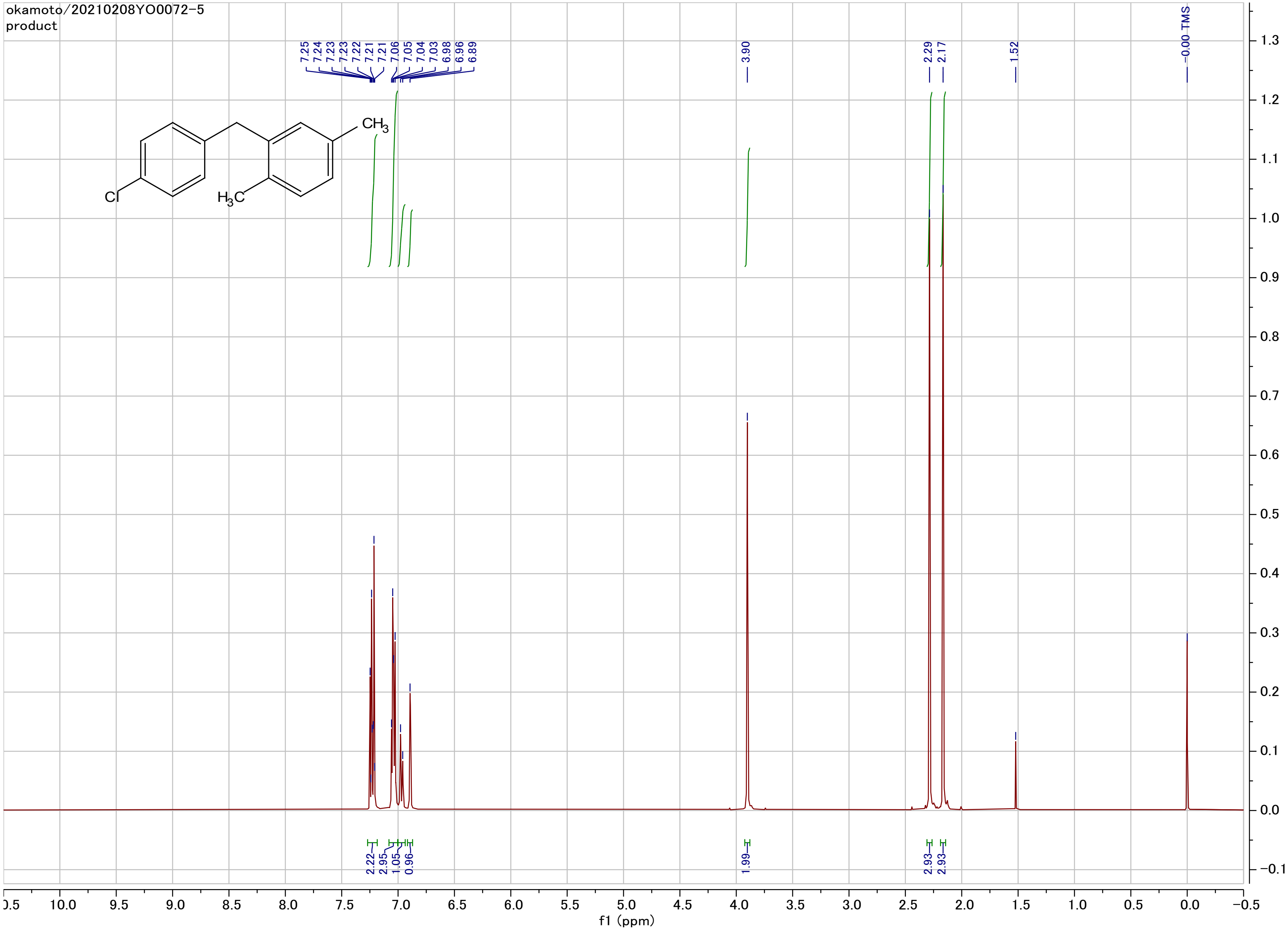

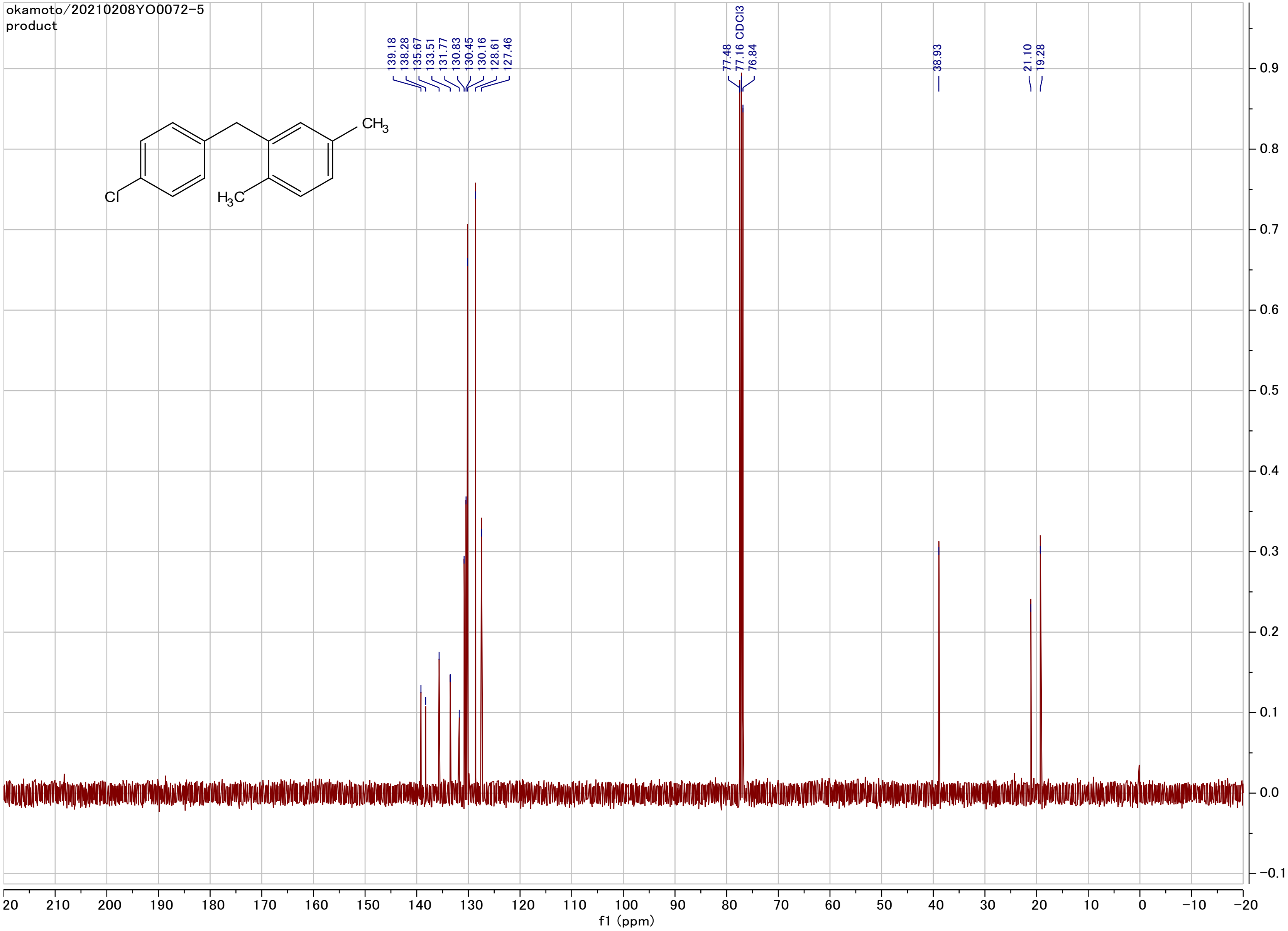
okamoto/20210208YO0072-5
product
139.18
138.28
135.67
133.51
131.77
130.83
130.45
130.16
128.61
127.46
77.48
77.16 CDCl3
76.84
38.93
21.10
19.28
CH3
Cl
H3C
f1 (ppm)

2-cyclohexyl-1,4-dimethylbenzene (**2j**)

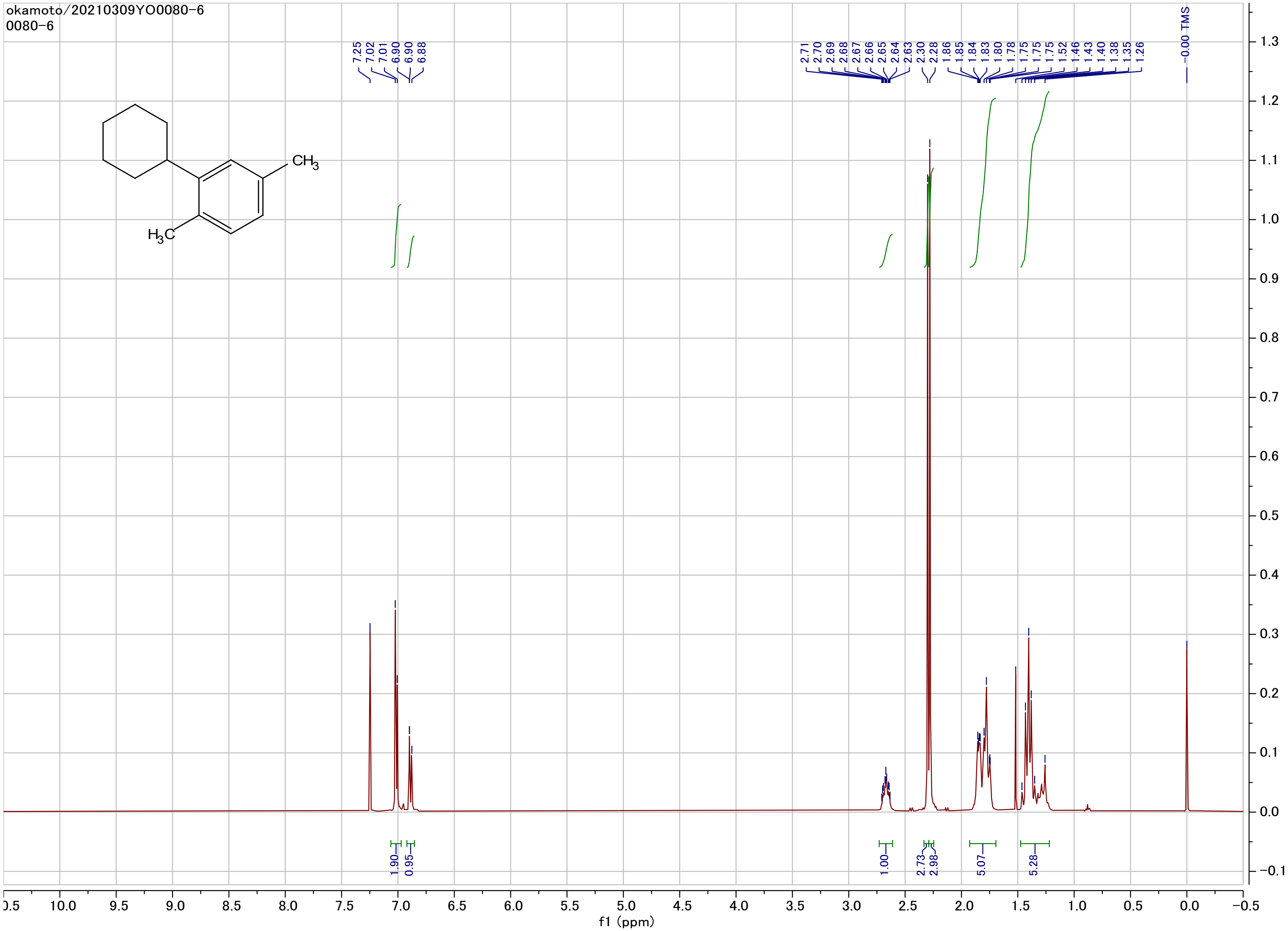

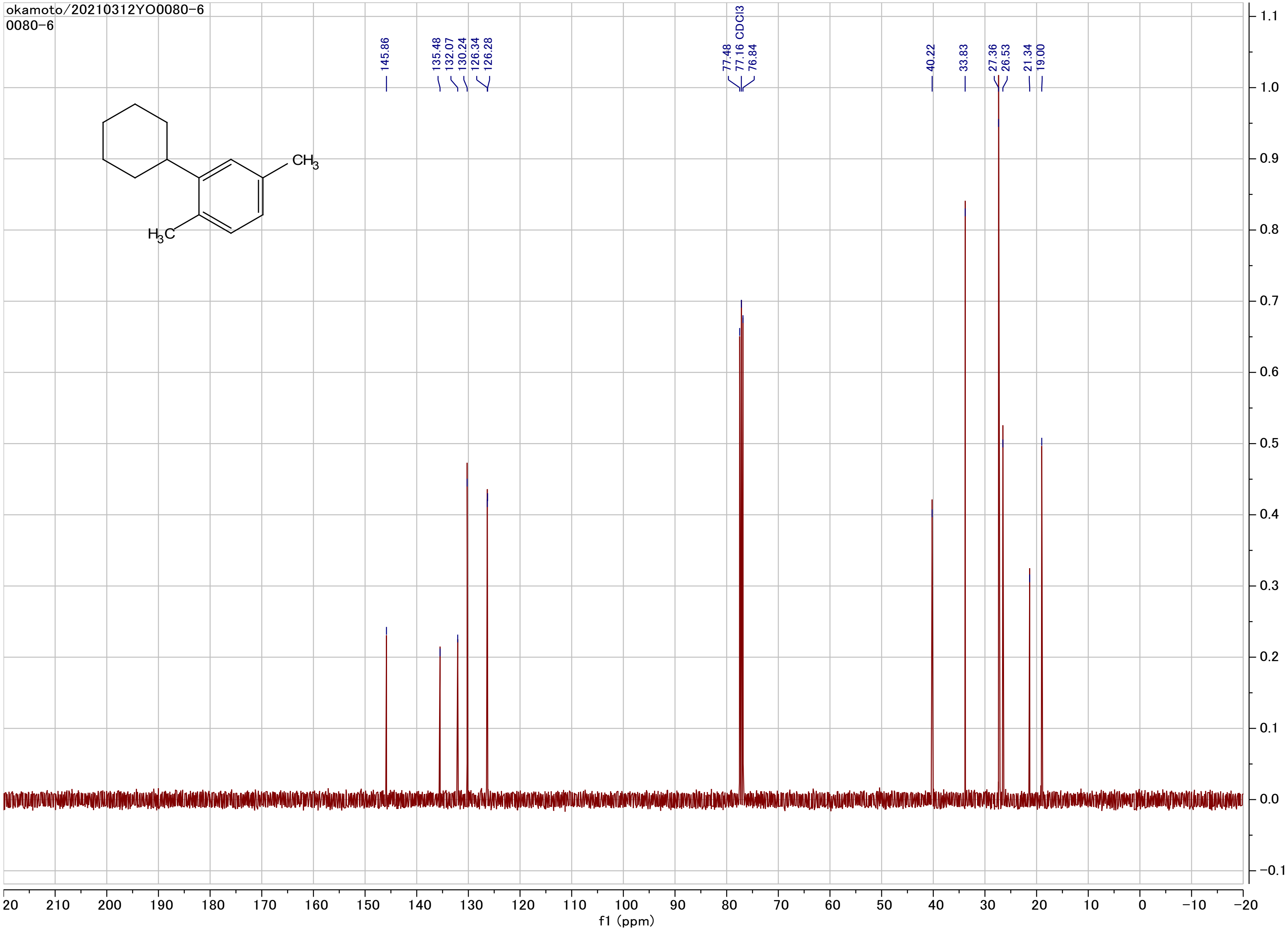
okamoto/20210312YO0080-6
0080-6
145.86
135.48
132.07
130.24
126.34
126.28
77.48
77.16 CDCl3
76.84
40.22
33.83
27.36
26.53
21.34
19.00
CH3
H3C
f1 (ppm)

1-(1-Adamantyl)-2,5-dimethylbenzene (**2k**)

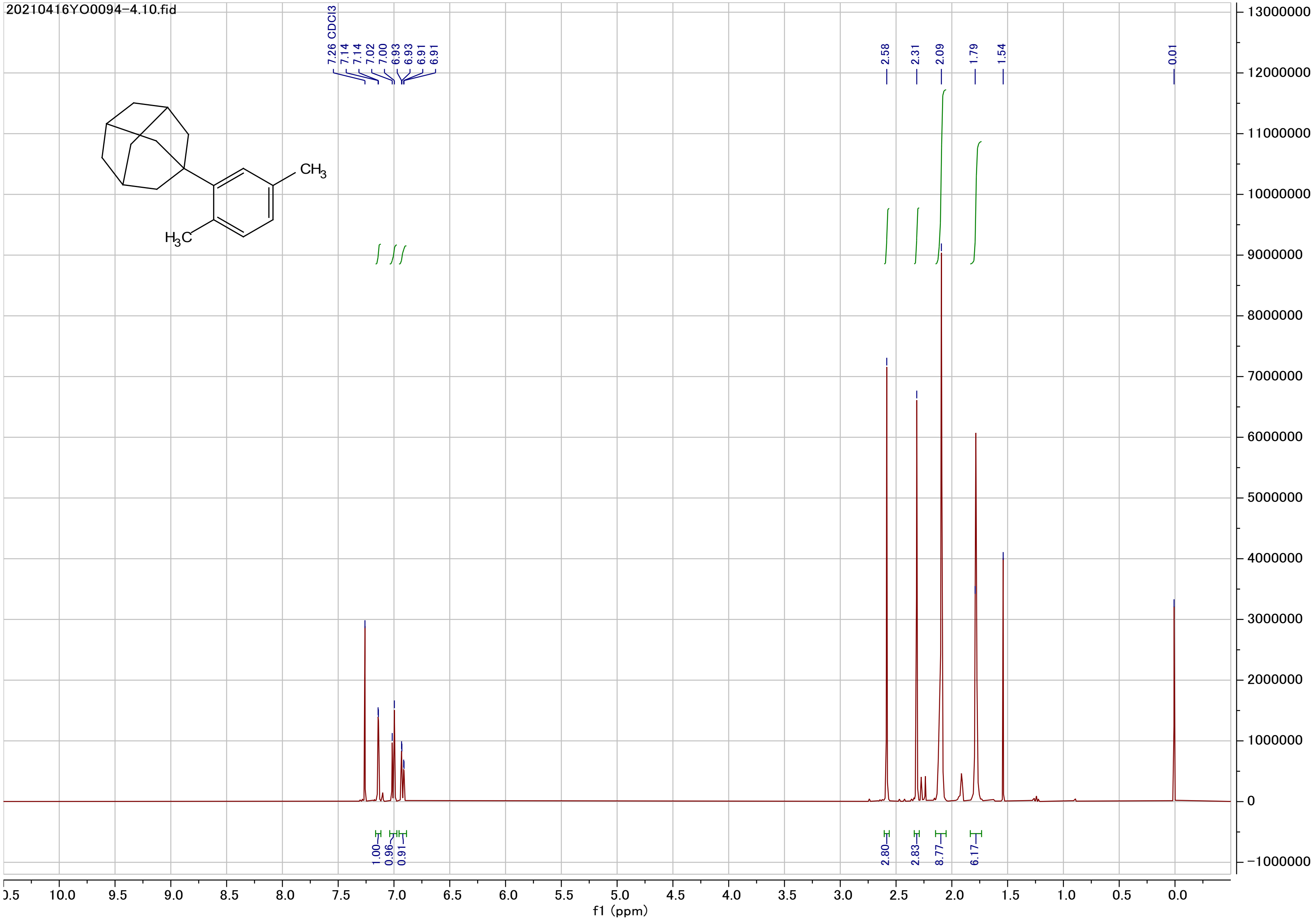

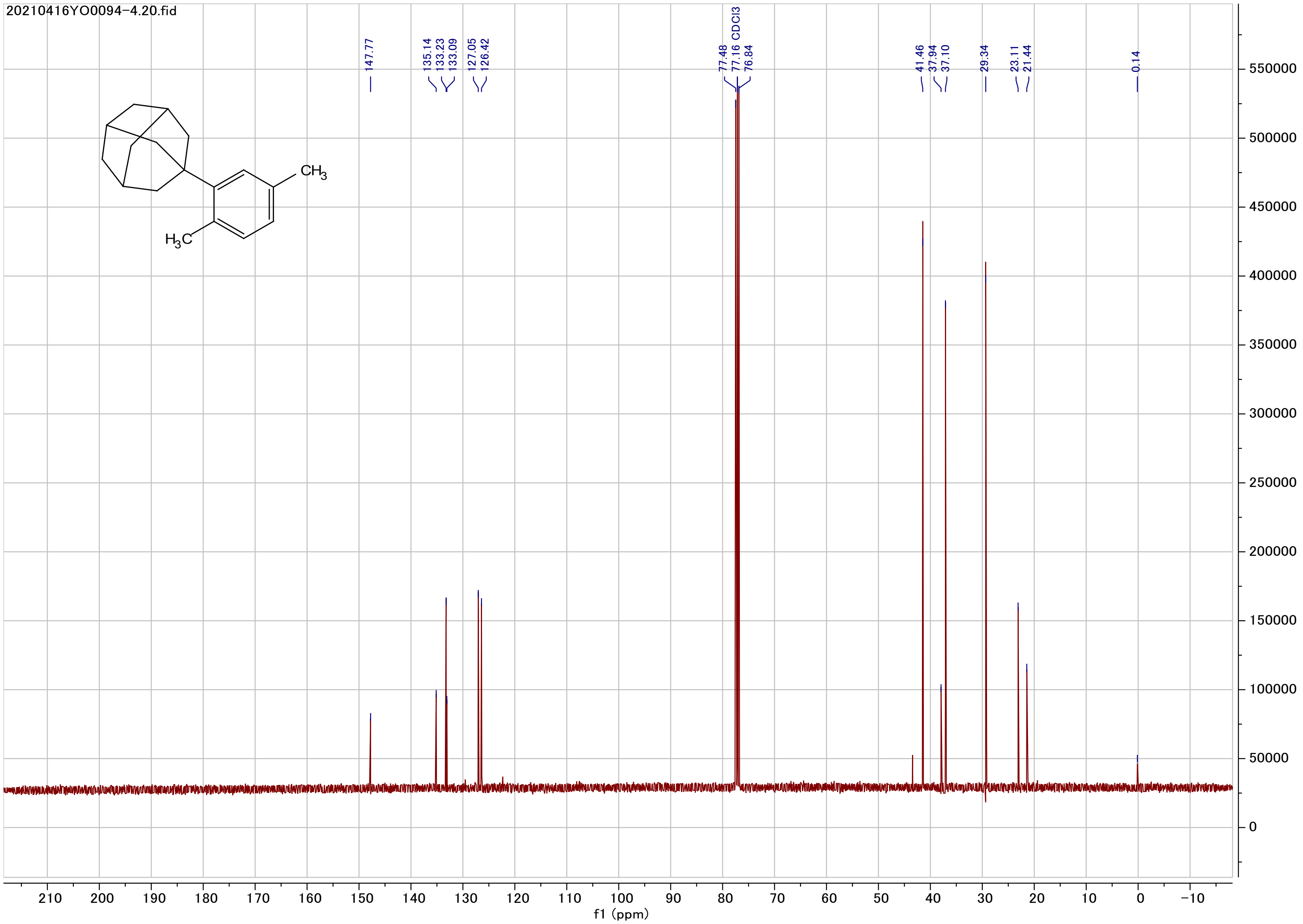
20210416YO0094-4.20.fid
147.77
135.14
133.23
133.09
127.05
126.42
77.48
77.16 CDCl3
76.84
41.46
37.94
37.10
29.34
23.11
21.44
0.14
CH3
H3C
f1 (ppm)
